# Mass Production of Customizable Core–Shell Active Materials in Seconds by Nano‐Vapor Deposition for Advancing Lithium Sulfur Battery

**DOI:** 10.1002/advs.202207584

**Published:** 2023-05-05

**Authors:** Lanxiang Feng, Zhiwei Zhu, Rui Yan, Xuewei Fu, Xuewei He, Dichen Wu, Hua Li, Zaiping Guo, Wei Yang, Yu Wang

**Affiliations:** ^1^ College of Polymer Science and Engineering Sichuan University Chengdu 610065 China; ^2^ School of Chemistry and Environment Southwest Minzu University Chengdu 610225 China; ^3^ School of Chemical Engineering & Advanced Materials The University of Adelaide Adelaide South Australia 5005 Australia

**Keywords:** core–shell active materials, high energy density lithium metal batteries, micro‐adhesion guided nanostorm technology, nano‐coating and nanofabrication, nano‐vapor deposition

## Abstract

Rational design and scalable production of core–shell sulfur‐rich active materials is vital for not only the practical success of future metal–sulfur batteries but also for a deep insight into the core–shell design for sulfur‐based electrochemistry. However, this is a big challenge mainly due to the lack of efficient strategy for realizing precisely controlled core–shell structures. Herein, by harnessing the frictional heating and dispersion capability of the nanostorm technology developed in the authors’ laboratory, it is surprisingly found that sulfur‐rich active particles can be coated with on‐demand shell nanomaterials in seconds. To understand the process, a micro‐adhesion guided nano‐vapor deposition (MAG‐NVD) working mechanism is proposed. Enabled by this technology, customizable nano‐shell is realized in a super‐efficient and solvent‐free way. Further, the different roles of shell characteristics in affecting the sulfur‐cathode electrochemical performance are discovered and clarified. Last, large‐scale production of calendaring‐compatible cathode with the optimized core–shell active materials is demonstrated, and a Li–S pouch‐cell with 453 Wh kg^−1^@0.65 Ah is also reported. The proposed nano‐vapor deposition may provide an attractive alternative to the well‐known physical and chemical vapor deposition technologies.

## Graphical abstract

A micro‐adhesion guided nano‐vapor deposition strategy is proposed to realize solvent‐free, scalable, and customizable production of core–shell sulfur‐rich active materials. Enabled by this nano‐vapor deposition technology, the electrochemical performance of sulfur cathode is further regulated by controlling the shell characteristics. This study may unlock the great potential of nano‐vapor deposition technology for the modification of active materials.

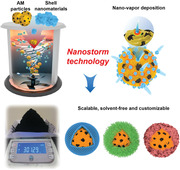

## 1 Introduction

Surface coating of electrode active materials (AM) is of great interest for addressing the critical issues coming with high‐voltage or high‐capacity of the AM particles, such as Ni‐rich/Li‐Mn‐rich cathodes and Si/Sn anodes.^[^ ^1^ ^]^ Rational design of the coating layer can effectively avoid the direct contact between AM and the electrolyte, while imparting various functions, such as buffering volume change, enhancing the electron/ion conductivity, scavenging intermediates of electrochemical reactions, and stabilizing electrode/electrolyte interphase.^[^ ^2^ ^]^ Especially for high capacity sulfur‐based cathode, it suffers from complicated electrochemistry and phase transition, such as repeating solid–liquid–solid conversion.^[^ ^3^ ^]^ The repeated dissolution‐deposition of AM during cycling generates tremendous sulfur intermediates (lithium polysulfides, LiPS), which gives rise to severe shuttle effect, uncontrollable LiPS deposition, notable volume change, and so on. Both shuttle effect and uneven LiPS deposition lead to irreversible sulfur loss and large volume change of AM that causes severe structural collapse and instability.^[^ ^4^ ^]^ Therefore, similar to traditional active materials, a functional coating layer is essential for encapsulating sulfur to resolve the above issues.^[^ ^5^ ^]^ To date, various surface coatings with multiple layers of graphene, carbon nanotube, metal/metal oxide nanoparticle, metal sulfides, nano‐carbon quantum dots, and polymer et.al.^[^ ^6^ ^]^ have been reported for the modification of sulfur AM. These coatings can trap LiPS by physical adsorption or chemisorption from certain polar groups (—C—O—, —N), allowing the electron transfer for converting LiPSs.^[^ ^7^ ^]^ However, in spite of the broad spectrum of coating materials reported, how the characteristics of the coatings such as electrical conductivity and sulfur‐philicity influence the Li—S electrochemistry, particularly, the adsorption, deposition, and conversion processes of S/LiPS, is not fully understood. This will hinder the design of advanced coating layers or core–shell structures for achieving high‐performance Li–S batteries.

To uncover the roles of coating layer characteristics in controlling the Li–S electrochemistry, a critical premise is to precisely control the coating structures (i.e., thickness and uniformity) for various types of coating nanomaterials. Only under this circumstance can the structure, electrical conductivity, and sulfur‐philicity of the coating layers be flexibly regulated. Thus far, solution^[^ ^8^ ^]^ and polymerization methods^[^ ^9^ ^]^ are broadly used to create various coating layers on sulfur AM surface, such as graphene@sulfur, polyaniline@sulfur, and hollow carbon@sulfur. These strategies can generate very beautiful and well‐controlled microstructures; however, they usually involve massive discharge of toxic solvents or complicated procedures, which are not industry‐friendly.^[^ ^2b^ ^]^ Other coating strategies, such as sputtering,^[^ ^10^ ^]^ chemical vapor deposition,^[^ ^11^ ^]^ electrostatic spray deposition,^[^ ^12^ ^]^ and atomic layer deposition^[^ ^13^ ^]^ are effective to achieve precisely controlled shell layers for core–shell AMs. However, the high costs, tedious operations, and low product yields remain a big challenge in practice. Consequently, there is an urgent demand on developing cost‐effective, scalable, and customizable coating technology.

**Figure 1:**
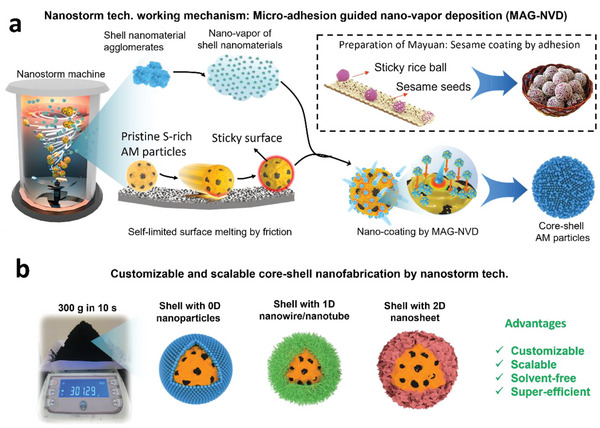
The concept of nanostorm technology with micro‐adhesion guided nano‐vapor deposition (MAG‐NVD) mechanism for the core–shell engineering of sulfur‐rich active materials. a) Schematic of MAG‐NVD nanostorm technology for superfast fabrication of core–shell S‐rich active particles. By taking the advantages of frictional heating and dispersion effects from high‐speed shearing, the S‐rich AM particles are transformed into sticky balls and the shell nanomaterial aggregates are broken into “nano‐vapor”. The micro‐adhesion guided deposition of “nano‐vapor” finally generates the nano‐coating, that is, the shell. Micro‐adhesion means the adhesion from the micro‐AM particle. b) Schematic of core–shell engineering by MAG‐NVD nanostorm technology for customizable and scalable production of advanced core–shell sulfur‐based active particles.

Herein, based on our previous work on nanostorm technology,^[^ ^14^ ^]^ we further propose a scalable, super‐efficient, micro‐adhesion guided nano‐vapor deposition (MAG‐NVD) nanostorm strategy to address the above demand. As illustrated in **Figure** **1**a, the MAG‐NVD nanostorm technology takes the full advantages of the effects from strong shearing and mixing. First, it can generate notable surface heating via friction, which can even melt the sulfur to create an adhesive surface for the pristine S‐rich AMs. Second, it can produce “nano‐vapor” from the shell nanomaterials via strong dispersion force. Third, the collision between the pristine AM particles and the “nano‐vapor” will also generate heat at the collision point. For the above effects, the pristine sulfur‐rich AM particles will be transformed into sticky balls, which help to catch the “nano‐vapor” and realize a superfast coating by nano‐vapor deposition. It should be noted that the MAG‐NVD nanostorm technology as illustrated by Figure 1a may not be limited by sulfur‐based active materials. There are several distinct advantages for the above MAG‐NVD strategy as illustrated in Figure 1b. First, the entire process is totally solvent‐free, extremely efficient, and easy to scale up. For instance, by the MAG‐NVD strategy, we have successfully prepared ≈300 g core–shell sulfur‐based particles in only 10 s, corresponding to a production rate of ≈30 g s^−1^ in laboratory scale. Second, one can flexibly design the coating layers with appropriate nanomaterials to achieve customized functions. Different from conventional studies on core–shell fabrication, this MAG‐NVD has the following merits. 1) It is totally solvent‐free, highly efficient, and even customizable. 2) It is effective for both small nanoparticles and big microparticles, such as the secondary S‐rich particles as introduced later. 3) It enables, for the first time, the studies on the relationship between the shell characteristics and the electrochemical performance of sulfur cathode. It is therefore believed that our study may provide not only a promising NVD technology to modify active particles with conductive coating but also instruction to the structure optimization of sulfur‐based active materials.

## 2 Results and Discussion

For the MAG‐NVD strategy, the nano‐vapor is an important factor to determine the uniformity and precision of shell nanomaterials. The nano‐vapor is formed by nanostorm strategy in home‐made machine. As shown in Figure S1 and Video S1, Supporting Information, we can obviously see the rising of nano‐vapor after 10 s dispersion in the nanostorm machine. Furthermore, the nano‐vapor is collected on the conductive resin with adhesion property. Compared with the pristine shell nanomaterial agglomerates (Figure S2a,b, Supporting Information), the nano‐vapor on the tape substrate presents a uniform nano‐scale dispersion (Figure S2c,d, Supporting Information). This result verifies the “nano‐vapor” generation capability of the nanostorm technology, which is one of the prerequisites for the next step of nano‐vapor deposition.

**Figure 2:**
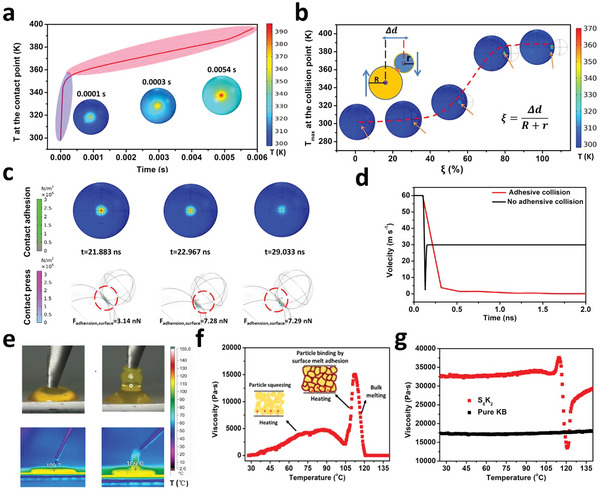
Simulation and experimentation studies on the micro‐adhesion guided nano‐vapor deposition (MAG‐NVD) mechanism. a) COMSOL simulation of the temperature change for particle surface by the friction between S‐rich AM particles and the metal wall of the nanostorm machine. b) COMSOL simulation of the temperature change for the AM particle surface by collision and friction with the shell nanomaterials. c) COMSOL simulation of the contact press and micro‐adhesion for the collision. d) Comparison of the velocity of nanocarbon particle after colliding with sulfur particle with/without adhesion by COMSOL simulation. e) Digital and thermal imager analysis of the sulfur melt adhesion with a carbon probe. f) Sulfur melting induced adhesion studied by thermo‐rheological testing of sulfur particles. g) Comparison of sulfur melting adhesion behavior for sulfur/carbon composite particles and pure carbon nanoparticles by thermo‐rheological testing.

In addition, for the MAG‐NVD strategy, the surface adhesion of the AM micro‐particles (simplified as micro‐adhesion) plays a critical role in the nano‐vapor deposition onto the S‐rich AM particles, that is, the formation of the core–shell S‐rich secondary particles. Usually, the heat generated by friction is viewed as an unfavorable factor during processing, which should be reduced as much as possible. Here, it is used to heat the S‐rich AM surface to achieve sticky melt. The friction‐based surface heating for the nanostorm strategy includes the friction with the chamber wall and the particle collision. Simulation of the friction‐based surface heating for S‐rich secondary particle was performed and studied using the finite element analysis by COMSOL multiphysics (see Experimental Section; Note S1, Supporting Information). As shown in **Figure** **2**a; Figure S3, Supporting Information, it was found that the friction heating between S‐rich AM particle and chamber wall raise the temperature at the friction site very significantly. Specifically, the temperature at the friction point changes from 298.15 to 398.56 K in only 6 ms (i.e., an increase of 100 K). The rapid rise of temperature by ≈50 K at the friction point is mainly due to the speed acceleration of the AM at the start of the friction. For the friction with constant shearing speed, the temperature rises up steadily with a much lower speed, which leads to ≈30 K change in 0.005 s. This temperature effect from steady friction should be understood as the primary reason for the surface heating as we started the shearing for only once. It should also be noted that the simulated friction was performed at a constant point of the AM particle by overlooking the rotation of the S‐rich AM particle. Meanwhile, the repeating of the friction for the same AM particle was not considered in the simulation for simplicity; although, it may happen for thousands of times. This simulation result indicates that the friction heating is super‐efficient to melt the particle surface. In addition, the collision friction between S‐rich AM particles and shell nanoparticles also generates hot spot at the surface as shown in Figure 2b; Figure S4, Supporting Information. It can be found that the degree of eccentricity for collision (see the insert in Figure 2b) remarkably impacts the friction heating effect. When the degree of eccentricity is above 50%, the friction heating effect increases rapidly due to the enhanced friction heat rate and friction time (see Figure S5, Supporting Information). When *ξ* is above 85%, one‐time collision friction can give rise to a maximum increase of 80 K at the collision point. In short, by the above simulation studies, the friction heating effects provide a super‐efficient strategy to melt the S‐rich AM surface, which will generate enhanced adhesion to the shell nanomaterials as will be discussed below.

The adhesion behavior between melt sulfur with shell nanoparticles is further studied by COMSOL multiphysics. As shown in Figure 2c, the heat contribution of nanoparticle collision onto the hot S‐rich AM particle after the friction with chamber wall is further simulated. The temperature overlying is obvious but the temperature at the collision point decreases very fast due to the heat dissipation. The contact adhesion behavior is studied for the particle–particle collision. It can be found that the adhesion force between sulfur melt and shell nanoparticle increases from an initial contact value of 3.14 to 7.28 nN after ≈7 ns. In Figure 2d, Figure S6, Supporting Information, the nanoparticle velocity before and after the collision is compared for the S‐rich AM particles with adhesive or non‐adhesive surface. One can find that the nanoparticle velocity changes from 60 to 30 m s^−1^ in the opposite direction, corresponding to an inelastic collision. In contrast, the nanoparticle velocity reduces sharply to zero for the collision with adhesive S‐rich AM particles, corresponding to a perfectly inelastic collision. To experimentally study the adhesion behavior of melt sulfur with carbon materials, sulfur solid blocks are melted into liquid at 130 °C (Figure S7, Supporting Information). As shown by Figure 2e, once there is a contact between the cool carbon needle and the sulfur melt surface, the sulfur melt adheres to the carbon needle and solidifies quickly due to heat dissipation, as shown by the digital photos and the temperature profiles collected by infrared thermal imager. The sticky surface is further evidenced by thermo‐rheological testing of the sulfur particles as shown by the shear viscosity versus temperature curve in Figure 2f. One can find that the sulfur particles experience volume expansion and particle squeezing at the first stage of heating, leading to an increase of viscosity (see Figure 2f, inset). When the temperature is close to the melting point of sulfur (≈110 °C), one can observe a significant increase in viscosity. This phenomenon should be the result of particle binding enabled by strong surface adhesion from sulfur melt.^[^ ^15^ ^]^ Furthermore, as shown in Figure 2g, the S_8_K_2_ mixture consisting of 20% KB and 80% sulfur experiences similar behavior to pure sulfur particles as revealed by the thermo‐rheological testing. For the pure KB, there is no significant change of viscosity during the heating process. The above results testify that the friction heat of S‐rich AM particles can generate stick surface, which helps to adhere carbon nanomaterials.

**Figure 3:**
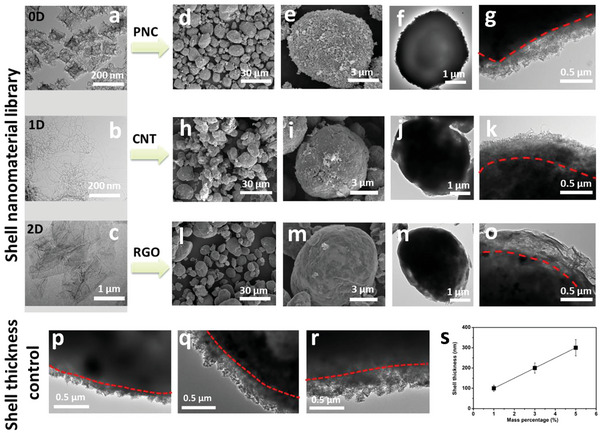
Demonstration of the customizable fabrication of core–shell AM particles by MAG‐NVD nanostorm tech. TEM images of a) PNC, b) CNT, and c) RGO. SEM images of d,e) PNC@SC particles; TEM images of f,g) PNC@SC particles; SEM images of h,i) CNT@SC particles; TEM images of j,k) CNT@SC particles; SEM images of l,m) RGO@SC particles; TEM images of n,o) RGO@SC particles. p–r) TEM images of the core–shell PNC@SC particles with different loading of PNC from 1 wt% (1PNC@SC), 3 wt% (3PNC@SC) to 5 wt% (5PNC@SC), respectively. s) Linear dependent behavior of the shell thickness on the mass loading of PNC shell nanoparticle.

To further take the advantages of melting sulfur with micro‐adhesion, one can control the characteristics of the shell layer by coating with different nanomaterials by MAG‐NVD strategy. This imparts the possibility to better understand the relationship between the structure‐properties of coating layers and the final electrochemical performance, which is significant for optimizing the core–shell structure but has been rarely investigated. In this work, we employed three representative building nano‐blocks for the coating, such as 0D PNC nanoparticles, 1D carbon nanotubes (CNTs) with an aspect ratio around 10, and 2D reduced graphene oxide (RGO), as shown by **Figure** **3**a–c; Figure S8, Supporting Information. These three kinds of carbon‐based nanomaterials have very different characteristics, including morphology, surface areas, and electrical conductivity. Specifically, the specific surface area critical for LiPSs trapping and the electronic conductivity critical for interface charge transfer are considered as the essential properties of the coating layer. Therefore, N_2_ adsorption isotherm testing was first performed and the result is shown in Figure S9a, Supporting Information. According to the IUPAC classification, PNC nanoparticles demonstrate type IV isotherm and type II hysteresis loop, indicating the presence of microspores and mesopores inside. Both CNT and RGO exhibit type II isotherm, indicating that there are no significant microporous or mesoporous structures.^[^ ^16^ ^]^ Furthermore, another critical factor of conductivity properties is collected by four‐point probe technique under varying pressure. RGO powder exhibits the highest conductivity over CNT and PNC powders, Figure S9b, Supporting Information. Figure S10, Supporting Information, shows that RGO possesses the highest conductivity (14 903 S m^−1^) at 6 MPa but the lowest surface area of 34 m^2^ g^−1^, in contrast to PNC which has the highest surface area (1119 m^2^ g^−1^) but the lowest electrical conductivity (32 S m^−1^). The CNT possesses the medium surface area (250 m^2^ g^−1^) and electrical conductivity (2350 S m^−1^).

To prepare the core–shell S‐rich electrode particles, the sulfur/carbon composite secondary particles (SC), as reported in our previous study,^[^ ^14^ ^]^ are employed as the core (Figure S11, Supporting Information). First, 0D cubic porous nano‐carbon (PNC) particle is used as the nanomaterial to build the coating layer. Specifically, the spherical S‐rich particle was prepared with sulfur content of 80 wt%. The PNC has plenty of micro‐/meso‐pores and a specific surface area ≈1100 m^2^ g^−1^ as shown in Figure S9, Supporting Information. The hierarchically porous structure and high surface area of PNC particles will benefit the adsorption and confinement of polysulfides to suppress the shuttle effect, which will be discussed in the following sections. From Figure 3d–g, one can observe that the PNC particles distribute uniformly on the SC core, which forms a core–shell configuration (PNC@SC). Furthermore, benefiting from the universality of the MAG‐NVD strategy, 1D CNT nanowire and 2D RGO nanoplate are successfully coated onto the SC core to form core–shell 5CNT@SC (Figure 3h–k) and 5RGO@SC (Figure 3l–o).

It is known that the thickness of coating layer is an important factor for the electrochemical performance of sulfur cathode, which has notable impacts on the ion‐/electron‐transfer and diffusion dynamics of polysulfides. To optimize the core–shell structure and gain a deeper understanding on the effect of coating layer on the electrochemical performance, a precise control of the coating thickness is necessary. It is revealed that, for the MAG‐NVD technology, the coating thickness can be facilely and effectively regulated by simply adjusting the precursor ratio during fabrication. As a result, one can control the coating thickness from 100 to ≈300 nm as shown in Figure 3p–r; Figure S12, Supporting Information. The transmission electron microscope (TEM) images in Figure S12, Supporting Information, further demonstrate that the PNC particles are uniformly bonded onto the surface of SC core. Specifically, this type of core–shell active material is prepared with different PNC‐coating loadings, that is, 1 wt%, 3 wt%, and 5 wt%. The obtained core–shell particles are designated as 1PNC@SC, 3PNC@SC, and 5PNC@SC, respectively. The coating thickness of 1PNC@SC, 3PNC@SC, and 5PNC@SC also shows an increasing thickness from 100 to 300 nm (Figure 3s). Besides, Figure S13, Supporting Information, shows that the average of diameter for 3PNC@SC particle is 7.108 µm, which is slightly larger than that for pure SC (6.864 µm). The energy‐dispersive X‐ray image and element mapping for a single 3PNC@SC particle show that S and C atoms are uniformly distributed and combined in the 3PNC@SC particle (Figure S14, Supporting Information). X‐ray diffraction (XRD) patterns in Figure S15, Supporting Information, show that pure SC and 3PNC@SC present the same diffraction peaks of sulfur. The sulfur content in 3PNC@SC is determined to be ≈77.8 wt% by thermo‐gravimetric analysis (TGA) in Figure S16, Supporting Information, which is consistent with the design of the compositions.

**Figure 4:**
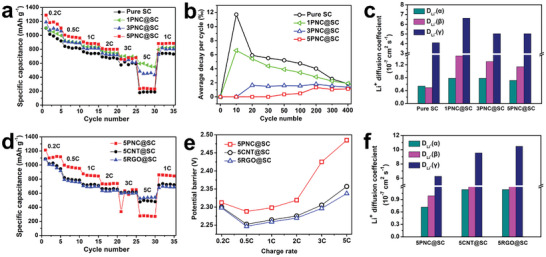
Uncovering the effects of shell characteristics on the sulfur electrochemical performance. a) Comparison of C‐rate performance of pure SC, 1PNC@SC, 3PNC@SC, and 5PNC@SC based electrodes. b) Comparison of average capacity decay per cycle for pure SC, 1PNC@SC, 3PNC@SC, and 5PNC@SC electrodes during cycling. c) Comparison of the Li‐ion diffusion coefficient of pure SC, 1PNC@SC, 3PNC@SC, and 5PNC@SC electrodes at peaks A, B, and C. d) Comparison of C‐rate performance of 5PNC@SC, 5CNT@SC, and 5RGO@SC electrodes. e) Comparison of the potential barrier for 5PNC@SC, 5CNT@SC, and 5RGO@SC electrodes during charging. f) Comparison of the Li‐ion diffusion coefficient of 5PNC@SC, 5CNT@SC, and 5RGO@SC electrodes at peaks A, B, and C.

The electrochemical performance of PNC@SC with various PNC coating thicknesses is further investigated in Li–S batteries. As shown in **Figure** **4**a; Figure S17, Supporting Information, all the PNC@SC cathodes exhibit a higher discharge capacity than that of the cathode with bare SC. This is because the porous PNC shell layer with high surface area effectively adsorbs and traps the soluble lithium polysulfides (LiPS) to reduce the sulfur loss and increase the sulfur utilization. When the current densities are less than 3 C, the 5PNC@SC cathode delivers the highest discharge capacities; namely, the capacity of 5PNC@SC cathode is 1289 mAh g^−1^ at 0.2 C (1 C = 1675 mAh g^−1^ for pure sulfur), in comparison to sulfur cathodes without shell structures (1105 mAh g^−1^), with 1PNC@SC shell (1108 mAh g^−1^) and 3PNC@SC shell (1190 mAh g^−1^), respectively. This 5PNC@SC sample also exhibits the best cycling stability at 0.2 C. However, it is worth noting that the rate capability deteriorates with the increase of coating thickness when further elevating the current density. Meanwhile, the capacity decay becomes obvious at an extremely high current density of 5 C. Specifically, the 5PNC@SC cathode only shows a discharge capacity of 230 mAh g^−1^, much lower than that of 1PNC@SC (595 mAh g^−1^) and 3PNC@SC (480 mAh g^−1^), but slightly higher than that of pure SC (190 mAh g^−1^) at a high current density of 5C. Compared with the cathode with bare SC particles, PNC@SC cathode with rational thickness of PNC coating does exhibit a better C‐rate capability due to the contribution of porous PNC shell.

To further understand the contribution of PNC coating to the electrochemical performance, the long‐term cycling stability at a moderate rate of 0.5 C is studied as shown in Figure S18, Supporting Information. The capacities at 400th show a downward trend from 5PNC@SC, 3PNC@SC, and 1PNC@SC to pure SC cathodes, which are 503, 447, 402, and 340 mAh g^−1^, respectively. To be more clear, the average decay rate of discharge capacity is compared at various cycling numbers as shown in Figure 4b. The results show that in the first ten cycles, the pure SC cathode suffers from a significant decay rate of 11.7%. This fast decay is likely due to the direct contact between SC particle surface and electrolyte, resulting in uncontrolled and severe sulfur loss during cycling. The average decay rate of capacity is greatly suppressed for the samples with the PNC shell or coating. Even for the 1PNC@SC cathode, the suppression of active material loss is obvious. Dramatically, for the 3PNC@SC and 5PNC@SC cathodes, they exhibit negligible capacity decay in the first ten cycles, indicating very strong polysulfide adsorption and confinement ability. More exciting, such a low capacity decay rate for 5PNC@SC electrode is held for ≈50 cycles. This result indicates that the PNC shell with a high surface area can effectively adsorb and confine the soluble LiPSs and inhibit the shuttle effect for Li–S batteries. As a result, after 400 cycles, the 5PNC@SC cathode displays an average decay per cycle of 0.11%, which is much lower than those of pure SC (0.16%), 1PNC@SC (0.15%), and 3PNC@SC (0.12%), respectively. It is noted that, after ≈20 cycles, SC and 1PNC@SC cathodes having weak LiPSs adsorption ability exhibit a decreasing tendency of decay rate with the increasing cycle number, which might be attributed to the generated Li_2_S*_n_* during cycling becoming a passive barrier impeding the further dissolving and escaping of LiPSs; and so, the shuttle effect. The average capacity decay rate of 3PNC@SC shows an obvious increase to 0.165% at the 20th cycle; and then, becomes almost steady afterward. By contrast, the decay rate of 5PNC@SC maintains to be the lowest along with the cycling, and it starts to increase after the 200th cycle. This result agrees with the above studies that the polysulfides’ capture ability increases with PNC coating thickness. The gradual capacity decay for 3PNC@SC and 5PNC@SC cathodes during cycling may result from uneven deposition and accumulation of Li_2_S*_n_* on the PNC surface, as well as the loss of active sulfur species, which will be investigated and discussed in details later.

To better understand the above electrochemical performance benefiting from the well‐controlled core–shell structure, we further perform visual–electrochemical study, differential scanning calorimetry (DSC), electrochemical impedance spectroscopy (EIS), and cyclic voltammetry (CV) testing. From Figure S19, Supporting Information, one can clearly see the obvious difference in the electrolyte color change between pure SC and PNC@SC cathodes. The transparent electrolyte becomes yellow once the pure SC cathode dips in the electrolyte due to the rapid sulfur dissolution even without cell charging/discharging. On the contrary, the electrolytes of PNC@SC cathodes remain colorless at the beginning, owing to the protection from the PNC shell. When the cells are discharged over 2 h, pure SC and 1PNC@SC experience an obvious color change of electrolyte, indicating that lots of LiPSs diffuse into the electrolyte. However, the diffusion of LiPSs is effectively suppressed for the electrodes with rational PNC coating thickness as revealed by 3PNC@SC and 5PNC@SC samples. The electrolyte is only slightly polluted by LiPSs even after 8 h of discharging. This result directly confirms the strong ability to adsorb and confine LiPSs by the PNC shell. The possible interaction between sulfur and carbon is further studied by DSC testing at a scanning rate of 10° C min^−1^. As shown in Figure S20, Supporting Information, PNC@SC cathodes present a much suppressed melting peak than that of pure SC, and 5PNC@SC shows the weakest melting peak. This result further reveals the strong nano‐confinement effects of high surface area PNC layer on the sulfur. The EIS results of fresh sulfur cathodes are shown in Figure S21, Supporting Information. It is found that the PNC shell plays an important role in facilitating the interfacial charge–transfer process. PNC@SCs show smaller charge–transfer resistance than that of pure SC. Specifically, 1PNC@SC has the smallest charge–transfer resistance (≈25 Ω), in comparison to SC (≈32 Ω), 3PNC@SC (≈27 Ω), and 5PNC@SC (≈32 Ω). This result suggests that there is possibly an optimal PNC coating thickness for achieving a low charge–transfer resistance and sufficient LiPS trapping capability, which is found ≈200 nm (i.e., the 3PNC@SC sample).

To further study and compare the Li‐ion diffusion, we conduct cyclic voltammetry measurement with various scanning rates ranging from 0.1 to 0.5 mV s^−1^ (Figure S22, Supporting Information). The Li‐ion diffusion capability is obtained according to the classical Randles–Sevcik equation,^[^ ^17^ ^]^

(1) $$I_{p}=\left(2.69\times 10^{5}\right)n^{1.5}AD_{\mathrm{Li}+}^{0.5}C\nu^{0.5}$$

where *I*_p_ is the peak current intensity, *n* is the charge transfer number per reaction species, *A* is the active electrode area (1.13 cm^−2^ in this work), D*_Li+_* is the Li‐ion diffusion coefficient, *C* represents the concentration of lithium ions, and *ν* is the scanning rate. To exclude the effect of electrode activation, all the CV plots of sulfur cathodes are collected from the second cycle. As shown in Figure S23, Supporting Information, all PNC@SCs show higher value than that of pure SC, which implies that PNC@SCs have faster Li‐ion diffusion. Furthermore, as shown in Figure 4c; Table S1, Supporting Information, the calculated Li^+^ diffusion coefficient value at peaks A, B, and C for PNC@SC cathode is much more than that of pure SC cathodes, and 1PNC@SC with thickest shell has the highest value of Li^+^ diffusion coefficient. These results are also consistent with the above EIS analysis. From the above characterizations and analysis, we can conclude that the PNC layer not only adsorbs LiPS to suppress the shuttle effect but also improves the Li‐ion diffusion. However, it should be noted that the interfacial electron‐transfer resistance inevitably increases with the increasing of coating thickness due to fair electron‐conductivity of PNC as will be shown later.

Furthermore, enabled by the above controlled characteristics of coating layer, the possible roles of shell characteristics in regulating the adsorption and deposition of polysulfides and electrochemical reaction kinetics can be studied in more details as shown below. For simplicity, only the same mass loading of 5 wt% was chosen for comparison. To do that, sulfur cathodes with 5PNC@SC, 5CNT@SC, and 5RGO@SC active particles are prepared and evaluated in coin‐cells. As shown in Figure 4d, at various current rates except 5C, 5PNC@SC cathode delivers the highest discharge capacity. For example, at a low current rate of 0.2C, 5PNC@SC cathode delivers a much higher discharge capacity of 1212 mAh g^−1^ and more stable cycling stability during the first five cycles than the other two cathodes (1100 mAh g^−^1 for 5CNT@SC and 1078 mAh g^−1^ for 5RGO@SC). However, the discharge capacity dramatically drops to 270 mAh g^−1^ at 5 C for the 5PNC@SC cathode. However, 5CNT@SC and 5RGO@SC cathodes deliver more stable and higher discharge capacities of 490 and 530 mAh g^−1^ at 5 C, respectively. Figure S24, Supporting Information, shows that 5RGO@SC cathode exhibits higher capacitance retention (50%) than that of 5CNT@SC cathode (45%) and 5PNC@SC cathode (22%) when the current density is increased from 0.2 C to 5 C. This result indicates that the 5RGO@SC has the best C‐rate performance, which is related to the RGO shell with best electrical transfer dynamic. Furthermore, from the galvanostatic discharge/charge profiles (Figure S25, Supporting Information), one can observe that all the cathodes show similar potential gap between discharging and charging plateaus at various current rates from 0.2 C to 2 C. However, at high current rates such as 3 C and 5 C, the discharge plateau of 5PNC@SC cathode disappears, while 5CNT@SC and 5RGO@SC cathodes exhibit well‐defined and flat discharge plateaus. The potential barrier during charging that represents the activation energy for transforming insulating Li_2_S_2_/Li_2_S to soluble LiPS,^[^ ^6d^ ^]^ is further analyzed at various current densities. As shown in Figure 4e; Figure S26, Supporting Information, the potential barrier of 5PNC@SC is significantly greater than that of 5CNT@SC or 5RGO@SC, indicating that 5PNC@SC has the most sluggish Li_2_S_2_/Li_2_S oxidation.^[^ ^3^, ^18^ ^]^ It undergoes a more obvious increase from 3 to 5 C. In contrast, 5RGO@SC yields the lowest potential barrier and weakest dependence on the C‐rate compared to 5PNC@SC and 5CNT@SC.

The above different C‐rate performances for three coating shells on sulfur‐rich AM particles are worthy of discussion. RGO and CNT have much higher electrical conductivity and much lower specific surface area (see Figure S10, Supporting Information) in comparison with PNC, and these characteristics of the coating layer may play a complicated role in controlling the C‐rate performance. Specifically, at a low C‐rate, high surface area of shell nanomaterials can adsorb the escaping LiPS as much as possible; thus, delivering the high sulfur utilization. Form the Figure S27, Supporting Information, we can observe that the electrolyte of 5CNT@SC and 5RGO@SC cathodes experiences an obvious color change in comparison with 5PNC@SC cathode. This phenomenon also implies that the confinement capability of LiPSs for PNC, CNT, and RGOs decreases in turn, which is consistent with the specific capacity of three types of core–shell cathodes at low C‐rate. However, the strong adsorption and fast accumulation of insulating LiPSs could degrade the electron transfer capability around the 5PNC@SC particles. Consequently, the electron transfer step becomes the dominating factor that limits the electrochemical reaction at high current rates. Besides, we conduct cyclic voltammetry measurement with various scanning rates (Figure S28, Supporting Information), and the linear fits of curves in Figure S29, Supporting Information, further show that the 5RGO@SC and 5CNT@SC exhibit greater slope than 5PNC@SC, indicating faster charge transfer around the S‐rich AM particles. As a result, the calculated Li^+^ diffusion coefficient value at peaks A, B, and C for 5RGO@SC cathode is much more than that of 5CNT@SC and 5PNC@SC cathodes, Figure 4f; Table S2, Supporting Information. This point is more clearly proved by the Nyquist plots as shown in Figure S30, Supporting Information. It shows that the charge–transfer resistance of 5PNC@SC, 5CNT@SC, and 5RGO@SC cathodes before cycling follows a downward trend from 33, 22, to 20 Ω, respectively.

To further study how the characteristics (mainly LiPS absorption and charge transfer capability) of the shell contribute to the sulfur electrochemical performance, it is important to investigate the cycling stability of the three different sulfur cathodes at various current rates of 0.5 C, 1 C, and 3 C. In this case, a relative low AM loading (2 mg cm^−2^) is employed for all the cathodes to minimize the contribution from other uncontrollable factors related to AM loading or thickness. At a small current density of 0.5 C, the 5PNC@SC cathode exhibits a lower decay rate of 0.1% than that of 5CNT@SC (0.12%) and 5RGO@SC (0.13%) as shown in Figure S31a, Supporting Information. When the current density is increased to 1 C (see Figure S31b, Supporting Information), 5RGO@SC and 5CNT@SC cathodes suffer from faster discharge capacity decay than that of 5PNC@SC cathode during the first 30 cycles. The average capacity decay for 5PNC@SC electrode is 0.18%, which is much lower than that of 5CNT@SC (0.28%) and 5RGO@SC (0.50%). However, after 200 cycles, the discharge capacity of 5RGO@SC cathode surpasses the 5CNT@SC and 5PNC@SC cathodes. The average capacity decay between 200 and 400 cycles for 5RGO@SC cathode is 0.083%, which is lower than that of 5CNT@SC (0.15%) and 5PNC@SC (0.16%). The merits of highly conductive shell, as demonstrated by RGO@SC and CNT@SC, become more predominant at a higher current density of 3C. As shown in Figure S31c, Supporting Information, after several cycles of activation (sulfur rearrangement), 5CNT@SC and 5RGO@SC deliver a similar discharge capacity of ≈700 mAh g^−1^@3C, which is much higher than that of 5PNC@SC (200 mAh g^−1^@3C).

**Figure 5:**
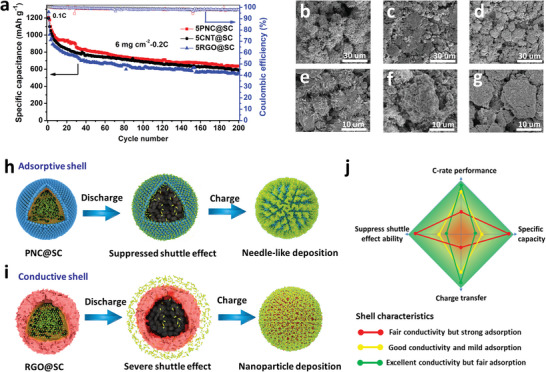
The effects of shell characteristics on the morphology evolution of the core–shell S‐rich particles after 200 cycles. a) Comparison of the cycling stability of 5PNC@SC, 5CNT@SC, and 5RGO@SC electrodes with a same sulfur loading of 6 mg cm^−2^. b–d) SEM images at low magnification for the cycled electrodes with 5PNC@SC, 5CNT@SC, and 5RGO@SC S‐rich particles, respectively. e–g) Surface morphology observation of the core–shell particles by SEM images at high magnification for the above cycled electrodes, respectively. Schematic of the different LiPSs diffusion and deposition behavior for S‐rich particles with h) strong absorptive but fair conductive shell and i) very conductive but fair absorptive shell. j) A summary of the contribution of shell characteristics to suppressing shuttle effect, charge transfer capability, high C‐rate performance, and specific capacity.

Furthermore, the merit of highly absorptive shell as demonstrated by PNC coating recovers at low current density even for an improved AM loading of 6 mg cm^−2^ as shown in **Figure** **5**a. In this case, the thick PNC@SC electrode exhibits the highest sulfur utilization and the best cycling stability at a low current density of 0.2 C. Besides, the analysis on the cycled sulfur cathodes is an important way to study the morphology evolution. As shown in Figure 5b–d, the core–shell particles are somehow compressed during cell assembling but basically sustain their particular morphology even after 200 cycles. To better investigate the structural stability, the core–shell particles are treated in a vacuum oven at a high temperature of 160 °C to remove sulfur as much as possible. As shown in Figure S32, Supporting Information, these particles still keep the spherical morphology even when most of the sulfur has been removed, which helps improve the structural stability during cycling. The EDS mapping of the cycled electrodes (Figure S33, Supporting Information) shows that the sulfur content in PNC@SC cathode is 16.9%, much higher than that of both CNT@SC (4.6%) and RGO@SC (3.1%) cathodes. The high sulfur content on the electrode surface should be the results of the strong trapping and significant accumulation of LiPS during cycling. A closer look at the surface reveals more details of the deposition morphology. From Figure 5e, one can clearly observe a thick layer of needle‐like structure on the surface of AM, which is believed to be the crystals Li_2_S*_x_* on the surface of cycled PNC@SC electrode. The thick Li_2_S*_x_* accumulation may deteriorate and even destroy the electron contact of the AM microenvironment due to its insulating nature, which is consistent with the electrochemical performance at high C‐rate. In contrast, both cycled 5CNT@SC (Figure 5f) and 5RGO@SC cathodes (Figure 5g) exhibit uniform Li_2_S*_x_* deposition in the form of nanoparticles. This result is further confirmed by the EIS testing of cycled cathodes as shown in Figure S34, Supporting Information. Compared with the CNT@SC and RGO@SC electrodes, the PNC@SC electrode after 200 cycles shows two semicircles. The second semicircles in the middle frequently represent the charge transfer resistance of cathode interface induced by the insulating of Li_2_S*_x_* agglomeration. This result indicates that PNC shell with high surface area and low conductivity can adsorb and accumulate a large amount of Li_2_S*_x_*, which is also confirmed by the SEM images (Figure 5b,e). The CNT and RGO shells with low surface area and excellent conductivity have weak capability of adsorbing Li_2_S*_x_*, while having strong capability of converting Li_2_S*_x_*. Therefore, the surfaces of CNT@SC and RGO@SC electrodes after cycling have small amounts of Li_2_S*_x_* agglomeration. The above results indicate that the characteristics of the coating layer play a critical role in regulating the AM deposition morphology, which may have significant influence on the cycling stability of the Li–S batteries.

The above findings indicate that the LiPS absorption and charge transfer capability of the shell play different roles in controlling the electrochemical performance, which has never been clarified to our best knowledge. Here, enabled by the on‐demand active material coating with the MAG‐NVD technology, we attempt to make them clear as discussed below. First, to better describe and understand the microstructures of the final sulfur cathode on composite level, we have proposed the concept of active material microenvironment that refers to the local environment surrounding individual AM particles.^[^ ^14^ ^]^ This active materials microenvironment includes the dry part (i.e., the local microstructures of conductive agent for electron conduction) and the wet part (i.e., the surrounding pore‐confined liquid electrolyte for ion conduction).

Based on the picture of AM microenvironment, it is proposed that the LiPS absorption and charge transfer capabilities of the shell will make a different contribution to the two parts of the microenvironment, as illustrated in Figure 5h. Specifically, compared with CNT and RGO, the PNC shell with high surface area and strong LiPSs trapping capability (see Figures S19 and S27, Supporting Information) can effectively suppress the diffusion of soluble LiPSs. The contribution of this strong trapping effect is multiple. First, it can help to protect the wet part of the microenvironment from serious LiPSs pollution, which significantly reduces the shuttle effects. As a result, the specific capacity is maintained at a high level because of the less loss of active materials during cycling, which is the main reason for the good cycle stability during the first few cycles. Second, the strong LiPS trapping capability coupled with the fair electrical conductivity of the PNC shell is not beneficial to build an efficient electron conductive microenvironment around the AM particles, which will limit the electron transfer kinetics. This situation becomes even more serious with the gradual accumulation of Li_2_S*_n_* passivation layer during repeated cycling. Therefore, for high C‐rates (i.e., 3 C and 5 C) or for long cycling testing, the 5PNC@SC cathode fails to maintain its superior performance at low C‐rates or the beginning as the charge–transfer capability becomes the dominating factor controlling the specific capacity.

For the shell with high electronic conductivity but fair LiPS trapping capability (the 5RGO@SC sample), the AM microenvironment experiences a very different story as illustrated in Figure 5i. First, due to its fair LiPS trapping capability, lots of LiPSs will escape from the inside of the AM particle and the wet part of the AM microenvironment will be seriously polluted. This uncontrolled diffusion will lead to serious shuttle effects, which are responsible for the much lower specific capacity and rapid capacity decay at low C‐rates (Figure 4d) as compared with 5PNC@SC. Second, the good electron conductivity of the CNT or RGO shell coupled with its fair LiPSs trapping capability is beneficial to build an efficient electron‐conduction microenvironment. In this case, shuttle effects become the dominating factor for the 5RGO@SC cathode. For this reason, the superior performance of 5RGO@SC or 5CNT@SC at high C‐rate of 5 C can be well explained as below. With the increasing of C‐rate, the conversion of dissolved LiPSs into Li_2_S or S speeds up as long as the transfer of electron is sufficient. This fast conversion gives less time to the diffusion of LiPSs and suppresses the shuttle effects. As a result, the specific capacity only decreases slightly with the increasing of C‐rate (Figure S24, Supporting Information).

To summarize and better understanding the above findings, we attempt to correlate the shell characteristics (the LiPS absorption and charge transfer capabilities as discussed in this study) with the electrochemical performance as illustrated in Figure 5j. Here, LiPS absorption and charge transfer capabilities are proposed as two key property factors determining the contribution of the shell or AM microenvironment to the final electrochemical performance of C‐rate performance and specific capacity. At low C‐rate or current density, the specific capacity is dominated by the shuttle effects because the charge–transfer capability is sufficient. Therefore, the core–shell AM with stronger LiPSs trapping capability (such as the case of 5PNC@SC) can deliver much higher capacity due to its suppressed shuttle effects, which leads to a higher conversion efficiency of LiPSs into the final AM product. At high C‐rate or current density, the specific capacity is dominated by the charge–transfer capability as the shuttle effects are self‐suppressed by a high current density, which speeds up the conversion process; and so, the conversion efficiency within a certain time. As a result, the core–shell AM with stronger charge–transfer capability (such as the case of 5RGO@SC) surpasses the performance of 5PNC@SC electrode at high C‐rate as found in Figure 4d; Figure S24, Supporting Information. These findings are very helpful for the design of S‐rich active materials as well the AM microenvironment.

**Figure 6:**
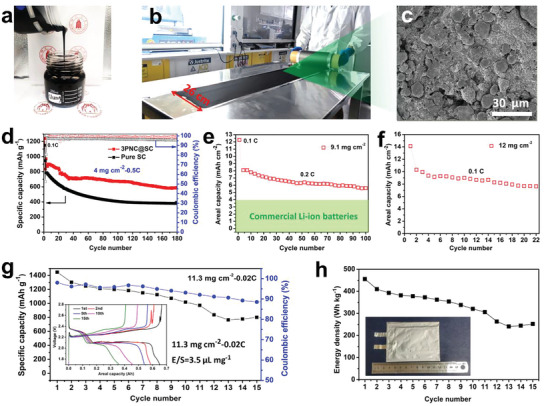
Scaling up the high‐performance calendaring‐compatible sulfur electrode with optimized 3PNC@SC core–shell particles. a) Digital photo of 3PNC@SC electrode slurry. b) Digital photo of the large‐scale preparation of 3PNC@SC electrode. c) SEM image of the 3PNC@SC electrode after calendaring at 1 MPa. d) Comparison of the cycle performance of 3PNC@SC and pure SC electrodes with a sulfur loading of 4.0 mg cm^−2^ at 0.5 C. e) Cycle performance of 3PNC@SC electrodes with a sulfur loading of 9.1 mg cm^−2^ at 0.2C. f) Cycle performance of 3PNC@SC electrode with a high sulfur loading of 12 mg cm^−2^ at 0.1 C. g) Cycling performance of 3PNC@SC electrode evaluated in Li—S pouch‐cell batteries; inset: the galvanostatic discharge/charge profiles. h) Energy density of the Li—S pouch cell constructed by 3PNC@SC electrode with E/S ratio of 3.5 µL mg^−1^ and two times extra lithium metal.

To exhibit the practicality of core–shell S‐rich secondary particles by MAG‐NVD strategy, we demonstrate the large‐scale production of sulfur cathode and further evaluate the electrochemical performance of high sulfur loading of cathode in lean electrolyte. **Figure** **6**a,b shows the large‐scale production of electrode slurry and coating cathode. After calendering at 1 MPa, the morphology of 3PNC@SC cathode is shown in Figure 6c. Meanwhile, the cathode porosity is also evaluated as shown in Figure S35, Supporting Information. Compared with the fresh cathode, the porosity of sulfur cathode after calendering at 1 MPa decreases from 73% to 48%. Furthermore, a medium sulfur loading of 4 mg cm^−2^ for pure SC and 3PNC@SC cathodes is further investigated in Figure 6d. After an activation process at 0.1 C, the 3PNC@SC cathode delivers a high discharge capacity ≈900 mAh g^−1^ at 0.5 C and shows good capacity retention of 68% over 180 cycles. The pure SC electrode shows a much lower discharge capacity and fast capacity decay from 786 to 380 mAh g^−1^ after 180 cycles, indicating a low sulfur utilization and severe shuttle effect. To surpass the commercial Li ion electrode, it is critical to develop thick sulfur cathodes and minimize the electrolyte/sulfur (E/S) ratio.^[^ ^19^ ^]^ Therefore, thick sulfur cathodes with 9.1 mg cm^−2^ sulfur loading are prepared and a low E/S ratio of 6 µL mg^−1^ is applied. Figure 6e shows that these thick electrodes deliver stable areal capacity of 6.5 mAh cm^−2^@0.1 C over 150 cycles for the electrodes with sulfur loading of 9.3 mg cm^−2^. To further increase the areal capacity, the sulfur loading is increased to 12 mg cm^−2^. As shown in Figure 6f, a high areal capacity of 14.2 mAh cm^−2^ at 0.5 mA cm^−2^ is delivered and a stable areal capacity of 9 mAh cm^−2^ at 0.1 C can be obtained over 22 cycles.

The above electrochemical performance in coin‐cell indicates that the PNC@SC electrode can deliver a high specific capacity and excellent cycling ability at a low current density. To further test this advantage in practical electrode, pouch cell with size of 4.7 cm × 7.7 cm is fabricated using a high sulfur loading of 11.3 mg cm^−2^‐0.02 C and a low E/S ratio of 3.5 µL mg^−1^. As shown in Figure 6g, the pouch‐cell delivers a high discharge specific capacity of 1447 mAh g^−1^ at 0.02 C, while exhibiting excellent cycling stability over only ten cycles. It is noticeable that the above electrochemical performance is obtained without any modification of binder, separator, and lithium anode. Therefore, as the cycle number increases, the pouch cell suffers from a low coulombic efficiency, which is mainly attributed to the severe anode corrosion. From the Figure 6g inset, the voltage curves of pouch‐cell demonstrate that discharge voltage is ≈2.1 V during the first ten cycles, indicating a low polarization and good health state of cell. Subsequently, the energy density of pouch‐cell is evaluated by a simplified model based on Equation (2). As shown in Figure 6h, the pouch‐cell delivers a high gravimetric energy density of 455 Wh kg^−1^ and good cycling stability. The results manifest the potential of the PNC@SC cathodes for practical high‐energy‐density Li–S batteries.

## 3 Conclusion

In summary, a versatile, scalable, and solvent‐free micro‐adhesion guided nano‐vapor deposition (MAG‐NVD) strategy is proposed for superfast fabrication of on‐demand core–shell sulfur‐rich particles. Such a MAG‐NVD strategy not only effectively controls the coating qualities (i.e., thickness and uniformity) for various types of coating nanomaterials but also possesses a super‐high efficiency and mass production capability without involving any solvent. Taking the advantages of this MAG‐NVD strategy, on‐demand coating of S‐rich particles is realized with different nanomaterials (from 0 to 2D). Enabled by the above well‐controlled core–shell AM, the different roles of shell characteristics in regulating the final electrochemical performance are systematically investigated for the first time, to our best knowledge. Particularly, for the shell with strong LiPSs trapping capability but fair electronic conductivity, the resultant electrode shows outstanding performance at low C‐rate but poor performance at high C‐rate. At the same time, it tends to generate big accumulation of Li_2_S*_x_* or thick passivation layer during repeated cycling, which could lead to fair cycling stability. On the contrary, for the shell with high charge‐transfer capability but very limited LiPS‐trapping capability, the opposite performance is found in comparison with the former case. Besides, to highlight the advantage in practical electrode, we prepare a large‐scale electrode and fabricate pouch‐cell with a high energy density of 455 Wh kg^−1^. In a word, these findings enabled by our MAG‐NVD strategy might provide new avenues to rational design and mass production of on‐demand core–shell S‐rich active materials, making an important step toward the practical success of high‐energy‐density Li–S batteries and beyond.

## 4 Experimental Section

### Materials

Sulfur (S) was bought from the Sigma–Aldrich Co. Ltd. Ketjen black (KB) and LA133 binder were purchased from HeFei KeJing Materials Technology Co. Ltd. Carbon nanotube (CNT) was purchased from ChengDu Organic Chemical Co. Ltd., CAS.

### Synthesis of 0D Porous Nano Carbon (PNC) and 2D Reduced Graphene Oxide (RGO)

PNC was synthesized with the method reported in the previous work.^[^ ^20^ ^]^ Specifically, 2‐Methylimidazole and colloidal silica were dissolved into deionized water to prepare a solution. Zn(NO_3_)_2_·6H_2_O and cetyltrimethyl ammonium bromide (CTAB) were pre‐dispersed and added into the above solution to synthesize ZIF‐8@SiO_2_ under magnetic stirring at 25 ^*ο*^C. Then, the ZIF‐8@SiO_2_ was pre‐carbonized under N_2_ flow at 1000 ^*ο*^C for 2 h, and further etched with NH_4_HF_2_. Last, the etched product was carbonized under the protection of Ar gas at 1000 ^*ο*^C for 5 h, in order to obtain the PNC. RGO was synthesized by thermally treating the graphene oxide (synthesized by a hummer's method^[^ ^21^ ^]^) under a N_2_ flow 1200 ^*ο*^C for 20 h.

### Preparation of Primary Sulfur/Carbon (Pure Sc) Secondary Particles, PNC@SC, CNT@SC, and RGO@SC Particles

Primary SC particles were prepared by a hail‐inspired nano‐storm technology reported in our previous work.^[^ ^14^, ^22^ ^]^ Specifically, 20 g KB was ground with 80 g sulfur powder for 10 min, and the pre‐mixture was transferred into a home‐made sealed stainless reactor with controlled temperature gradient and high‐speed agitator blade to form pure SC. Furthermore, 1 g PNC and 99 g pure SC were transferred into the above home‐made instrument in 80 °C for high speed stirring. The speed rate was controlled in a speed ranging from ≈2000–30 000 r min^−1^. After 10 s, the PNC@SC particles with a PNC weight ratio of 1% were obtained, which was named as 1PNC@SC. Accordingly, 3PNC@SC and 5PNC@SC particles were prepared by changing the precursor mass ratio. For example, 5PNC@SC particle was fabricated in precursor mass ratio of 5 wt% of PNC and 95 wt% of pure SC. For 5CNT@SC and 5RGO@SC particles, PNC precursor was replaced with CNT and RGO, and the stirring time was increased to 30 s and 1 min, respectively.

### Fabrication of Pure SC, PNC@SC, CNT@SC, and RGO@SC Electrodes

PNC@SC particle was mixed with the commercially conductive additive CNT and binder LA133 in a mass ratio of 80:10:10 to prepare the electrode slurry. Subsequently, the electrode slurry was coated on the Al foil and vacuum dried at 60 °C for 10 h to obtain the PNC@SC electrode. For comparison, pure SC, CNT@SC, and RGO@SC electrodes were prepared with same precursor mass ratio and procedure treatments.

### Materials Characterization

The morphologies were studied by SEM (FESEM, Japan) with energy‐dispersive X‐ray analysis (EDX) and TEM (JOEL2010F). The sulfur/carbon binding interaction of various samples was investigated and compared by differential scanning calorimetry (DSC) Q20 (TA Instruments, Milford, MA, USA) under a nitrogen atmosphere at the scan rate of 10 °C min^−1^. The surface area for various precursors was measured by nitrogen adsorption/desorption isothermal (ASAP 2020 HD Analyzer). Thermogravimetric analyses (TGA, Q600, TA instrument, USA) were used to obtain the sulfur content under a nitrogen atmosphere at a heating rate of 10 °C min^−1^ from 50 °C to 800 °C. The electronic conductivity was collected by first pressing shell powders into a platelet under varying pressures and then testing platelet with a four‐point probe electrical system (ST2722, Tongde instrument, China). Electrode porosity was evaluated by the Equation (2), where m1 and m2 represent the weight of n‐butanol and electrode, and 𝜌1and 𝜌2 are the density of n‐butanol and electrode, respectively.

(2) $$\mathrm{Electrode}\;\mathrm{Porosity}\;=\frac{m1/\rho 1}{m1/\rho 1+m2/\rho 2}\;\;\times 100\%$$

### Electrochemical Characterization

Coin‐cells (CR2032) were fabricated in an argon‐filled glove‐box using variously prepared cathodes, Li metal anode, and the Celgard 2325 separator. For the pouch‐cells, the size of cathodes was 7.7 cm × 4.7 cm. The electrolyte used was 1 m lithium bis(trifluoromethanesulfonyl) imide (LiTFSI) dissolved in a mixture of 1,3‐dioxolane and dimethoxyethane (1:1 in volume) with 1% addition of LiNiO3 additive. All cells were performed and compared by Neware Battery system with a voltage ranging from 1.7 to 2.8 V (vs Li/Li^+^) at 25 °C. Cyclic voltammetry (CV) and electrochemical impedance spectroscopy (EIS) were conducted by an electrochemical workstation (ParStat400, USA). CV measurements were performed in a voltage range of 1.5–3 V at various scanning rates from 0.1 to 0.5 mV s^−1^. The Nyquist plots of the sulfur cathodes were recorded by electrochemical workstation in the frequency range of 10 mHz–100 kHz with an amplitude of 5 mV at an open circuit.

The evaluation of energy density (*E_g_*) was conducted by the following method. The *E*_g_ were calculated from Equation (3). *E*_g_ is the gravimetric energy density (Wh kg^−1^). *V* is the output voltage of pouch‐cell (≈2.1 V). *C* is the areal capacity of pouch cell (mAh cm^−2^). Mi and Ti are the mass per unit square (mg cm^−2^) of the whole pouch cell without consideration of the Aluminum soft packaging film. Specifically, the pouch cell components contained a single layer of compressed cathode (S‐loading was 11.3 mg cm^−2^), an assumed anode with 2 × Li excess, separator (*ρ* was ≈ 0.95 g cm^−3^), current collector (*ρ* was ≈ 2.7 g cm^−3^), and electrolyte (*E*/*S* = 3.5 µL mg^−1^, *ρ* was ≈ 1.2 g cm^−3^).

(3) $$Eg\;=\frac{VC}{\sum Mi}$$

### Simulation

The commercial finite element package COMOSL was performed to investigate the theoretical calculation using the coupled 3D unsteady heat equations (see Note S1, Supporting Information). Specifically, based on the heat conduction equation, the value of heat source generated by collision and sliding friction was used to compute the surface temperature distribution. Before calculation, some assumptions were taken into consideration, such as low convection heat transfer, no gravity, and single point friction. For the adhesion effect during the collision, the distance between master face (carbon) and slave face (sulfur) was monitored to investigate, and the tendency of temperature and adhesion were further computed.

In the present work, the speed of particle motion was 60 m s^−1^, which was determined by the linear speed of home‐made nanostorm machine. The diameters of sulfur particle and carbon particle were set to 1000 and 400 nm, respectively. The medium of natural convection in the simulation process was air. The initial temperature was 298.15k.

## Conflict of Interest

The authors declare no conflict of interest.

## Supporting information

Supporting Information

Supplemental Video 1

## Data Availability

The data that support the findings of this study are available on request from the corresponding author. The data are not publicly available due to privacy or ethical restrictions.

## References

[1] a) G.‐L. Xu , Q. Liu , K. K. S. Lau , Y. Liu , X. Liu , H. Gao , X. Zhou , M. Zhuang , Y. Ren , J. Li , M. Shao , M. Ouyang , F. Pan , Z. Chen , K. Amine , G. Chen , Nat. Energy 2019, 4, 484;

[2] a) P. Yan , J. Zheng , J. Liu , B. Wang , X. Cheng , Y. Zhang , X. Sun , C. Wang , J. G. Zhang , Nat. Energy 2018, 3, 600;

[3] a) H. Pan , X. Wei , W. A. Henderson , Y. Shao , J. Chen , P. Bhattacharya , J. Xiao , J. Liu , Adv. Energy Mater. 2015, 5, 1500113;

[4] H. Pan , J. Chen , R. Cao , V. Murugesan , N. N. Rajput , K. S. Han , K. Persson , L. Estevez , M. H. Engelhard , J.‐G. Zhang , K. T. Mueller , Y. Cui , Y. Shao , J. Liu , Nat. Energy 2017, 2, 813.

[5] a) J. Zhang , H. Ye , Y. Yin , Y. Guo , J. Energy Chem. 2014, 23, 308;

[6] a) M. Yu , R. Li , M. Wu , G. Shi , Energy Storage Mater. 2015, 1, 51;

[7] a) L. Feng , P. Yu , X. Fu , Z. M. Zhang , K. Davey , Y. Wang , Z. Guo , W. Yang , ACS Nano 2022, 16, 7982; 35486450 10.1021/acsnano.2c00882

[8] a) P. Yu , L. X. Feng , D. C. Ma , X. R. Sun , J. K. Pei , X. J. Zha , R. Y. Bao , Y. Wang , M. B. Yang , Z. P. Guo , W. Yang , Adv. Funct. Mater. 2021, 31, 2008652;

[9] a) W. Li , Q. Zhang , G. Zheng , Z. W. Seh , H. Yao , Y. Cui , Nano Lett. 2013, 13, 5534; 24127640 10.1021/nl403130h

[10] Y. Ma , L. Li , J. Qian , W. Qu , R. Luo , F. Wu , R. Chen , Energy Storage Mater. 2021, 39, 203.

[11] J. L. Shi , C. Tang , H. J. Peng , L. Zhu , X. B. Cheng , J. Q. Huang , W. Zhu , Q. Zhang , Small 2015, 11, 5243. 26265205 10.1002/smll.201501467

[12] C. Zhu , Y. Fu , Y. Yu , Adv. Mater. 2019, 31, 1803408. 10.1002/adma.201803408 30302831

[13] X. Meng , X. Q. Yang , X. Sun , Adv. Mater. 2012, 24, 3589. 22700328 10.1002/adma.201200397

[14] L. Feng , Y. Ji , Z. Zhu , P. Yu , X. Fu , M. Yang , Y. Wang , W. Yang , Energy Storage Mater. 2021, 40, 415.

[15] Y. Wang , L. Chen , L. Scudiero , W. H. Zhong , Chem. Commun. 2015, 51, 15967. 10.1039/c5cc06524k 26383233

[16] a) C. Fan , V. Nguyen , Y. Zeng , P. Phadungbut , T. Horikawa , D. D. Do , D. Nicholson , Microporous Mesoporous Mater. 2015, 209, 79;

[17] S. Y. Bai , X. Z. Liu , K. Zhu , S. C. Wu , H. S. Zhou , Nat. Energy 2016, 1, 16094.

[18] Y. Yang , G. Zheng , S. Misra , J. Nelson , M. F. Toney , Y. Cui , J. Am. Chem. Soc. 2012, 134, 15387. 22909273 10.1021/ja3052206

[19] R. Fang , S. Zhao , Z. Sun , D. W. Wang , H. M. Cheng , F. Li , Adv. Mater. 2017, 29, 1606823. 10.1002/adma.201606823 28380284

[20] C. Wu , Q. Liu , R. Chen , J. Liu , H. Zhang , R. Li , K. Takahashi , P. Liu , J. Wang , ACS Appl. Mater. Interfaces 2017, 9, 11106. 28264161 10.1021/acsami.6b16848

[21] W. S. Hummers , R. E. Offeman , J. Am. Chem. Soc. 1958, 80, 1339.

[22] L. Feng , Z. Zhu , Y. He , Y. Ji , X. He , L. Jing , M. Yang , W. Yang , Y. Wang , J. Energy Chem. 2022, 65, 565.

