# Supplementary material for: Mass Production of Customizable Core–Shell Active Materials in Seconds by Nano‐Vapor Deposition for Advancing Lithium Sulfur Battery
Source: Adv Sci (Weinh). 2023 May 5;10(20):2207584. doi: 10.1002/advs.202207584 (PMC10369239; doi:10.1002/advs.202207584)
Supplement: Supplementary file 1 — Supporting Information [file ADVS-10-2207584-s002.pdf]

ADVANCED SCIENCE
Open Access

Supporting Information

for *Adv. Sci.*, DOI 10.1002/advs.202207584

Mass Production of Customizable Core–Shell Active Materials in Seconds by Nano-Vapor Deposition for Advancing Lithium Sulfur Battery

Lanxiang Feng, Zhiwei Zhu, Rui Yan, Xuewei Fu, Xuewei He, Dichen Wu, Hua Li, Zaiping Guo, Wei Yang* and Yu Wang**

Supplementary Information

Mass Production of Customizable Core-Shell Active Materials in Seconds by Nano-Vapor Deposition for Advancing Lithium Sulfur Battery

Lanxiang Feng[1,2,⊥], Zhiwei Zhu[1,⊥], Rui Yan[1], Xuewei Fu[1], Xuewei He[1], Dichen Wu[1], Hua Li[1], Zaiping Guo[3]*, Wei Yang[1]* and Yu Wang[1]*

[1] College of Polymer Science and Engineering, Sichuan University, Chengdu 610065 Sichuan, China

[2] School of Chemistry and Environment, Southwest Minzu University, Chengdu 610225 Sichuan, China

[3] School of Chemical Engineering & Advanced Materials, The University of Adelaide, Adelaide, SA 5005, Australia

[⊥] These authors contributed to this work equally.

* Corresponding authors:

Zaiping Guo; Email: zaiping.guo@adelaide.edu.au

Wei Yang; Email: weiyang@ scu.edu.cn

Yu Wang; Email: yu.wang3@ scu.edu.cn

Supplementary Note

The commercial finite element software package COMSOL Multiphysics is used to couple the solid mechanics and heat transfer interface. Three-dimensional unsteady heat conduction equation and friction are selected to theoretically study the synergistic effects of impact, friction and heat generation in MAG-NVD nanostorm technology.

The governing equation of the three-dimensional transient nonlinear heat conduction problem with temperature-dependent thermophysical properties and a heat source can be described by Formula (1)[1].

$$\rho c_p \frac{\partial T}{\partial t} - k\Delta T = Q \qquad (1)$$

Where ρ is material density, c_p is constant pressure heat capacity of materials, k represents thermal conductivity, Q is Additional heat source.

The additional heat source in the simulation comes from friction in MAG-NVD nanostrom technology. Considering convection effect and material thermoelastic damping, the unsteady heat conduction process of solid surface caused by a heat source. Additional heat source Q is expressed by Formula (2).

$$Q = \varphi f_s \frac{\partial \boldsymbol{u}}{\partial t} - \alpha T{:}\frac{d\boldsymbol{S}}{dt} + \rho c_p \boldsymbol{u_{flux}} \cdot \nabla T \qquad (2)$$

Where $\boldsymbol{u_{flux}}$is thermal convection velocity ($\boldsymbol{u_{flux}}$=0.1 m/s), c_p is constant pressure heat capacity of materials, α is thermal expansion coefficient, $\boldsymbol{S}$ is second Piola-Kirchhoff tensor, φ is heat distribution coefficient ($\varphi = 0.5$) in default, f_s is frictional stress ($\text{friction coefficient} = 0.31$). $\boldsymbol{u}$ is particle movement displacement ($u = 60\ m\ s^{-1}$) in model. $\varphi f_s \frac{\partial \boldsymbol{u}}{\partial t}$ is the friction heat generation term, and the $\alpha T{:}\frac{d\boldsymbol{S}}{dt}$ and $\rho c_p \boldsymbol{u_{flux}} \cdot \nabla T$ refer to the material thermal expansion term and convection heat transfer term, respectively.

Friction stress can be calculated by the equilibrium equations for solid mechanics

which are given by Newton's second law[2].

$$\rho \frac{\partial^2 \boldsymbol{u}}{\partial t^2} + \lambda \frac{\partial \boldsymbol{u}}{\partial t} - \nabla \cdot \sigma = f_v \qquad (3)$$

Where ρ is material density, $\boldsymbol{u}$ is particle movement displacement, λ is damping coefficient, σ is stress tensor, f_v is volume force. $\lambda \frac{\partial \boldsymbol{u}}{\partial t}$ is the damping term. Regardless of the adhesion process, $\lambda \frac{\partial \boldsymbol{u}}{\partial t} = f_s + f_p$, and f_p is contact pressure. when adhesion is taken into account, $\lambda \frac{\partial \boldsymbol{u}}{\partial t} = f_s + f_p + f_a$. The body force only contains the inertial term because the simulation mainly studies the micro contact process.

When it refers to the adhesion, the adhesion formulation in the software defines an incremental displacement jump $\boldsymbol{u}$ in the local boundary system as described by Formula 4. As shown in below Formula 4, we use the effective gap distance h_n and the slip $\boldsymbol{h}_t$.

$$\boldsymbol{u} = \{0{,}0, \langle h_n \rangle\} + \boldsymbol{T}_b^{-T} \cdot \Delta \boldsymbol{h}_t \qquad (4)$$

$$f_a = \boldsymbol{ku} \qquad (5)$$

The adhesion starts to act when the temperature reaches the melting point of sulfur and the $h_n < 0$, and the displacement can be achieved according the Formula 4. As a result, we can get f_a from Formula 5, where $\boldsymbol{k}$ is the adhesive stiffness and its value is defined based on the bulk modulus and shear modulus of sulfur melt.

During the simulation of collision and friction, the full coupling method is used to solve the temperature, velocity, displacement and other variables according to the above equations.

Supplementary Figures

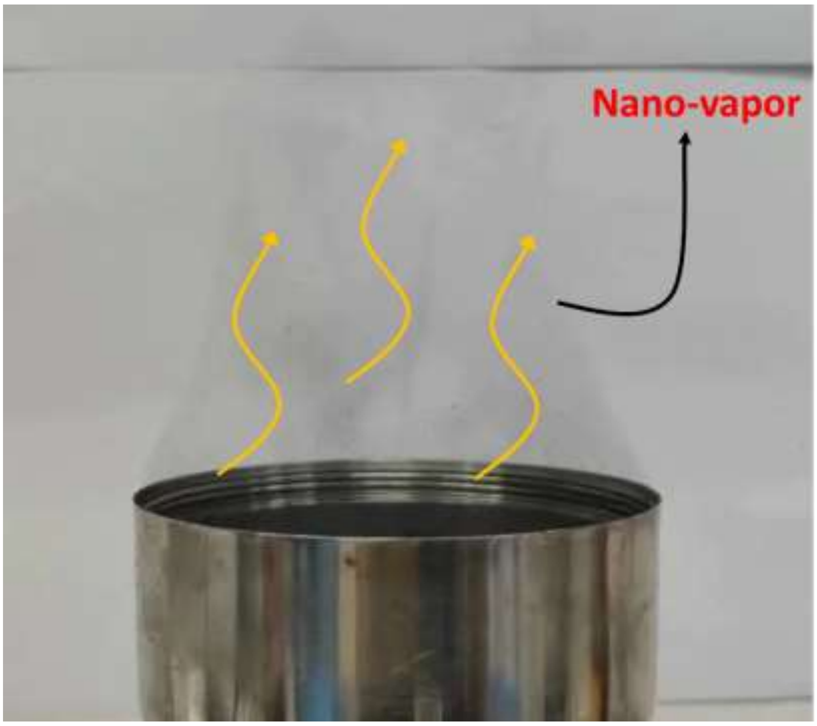

Figure S1. Digital photo of the nano-vapor produced by the nanostorm technology.

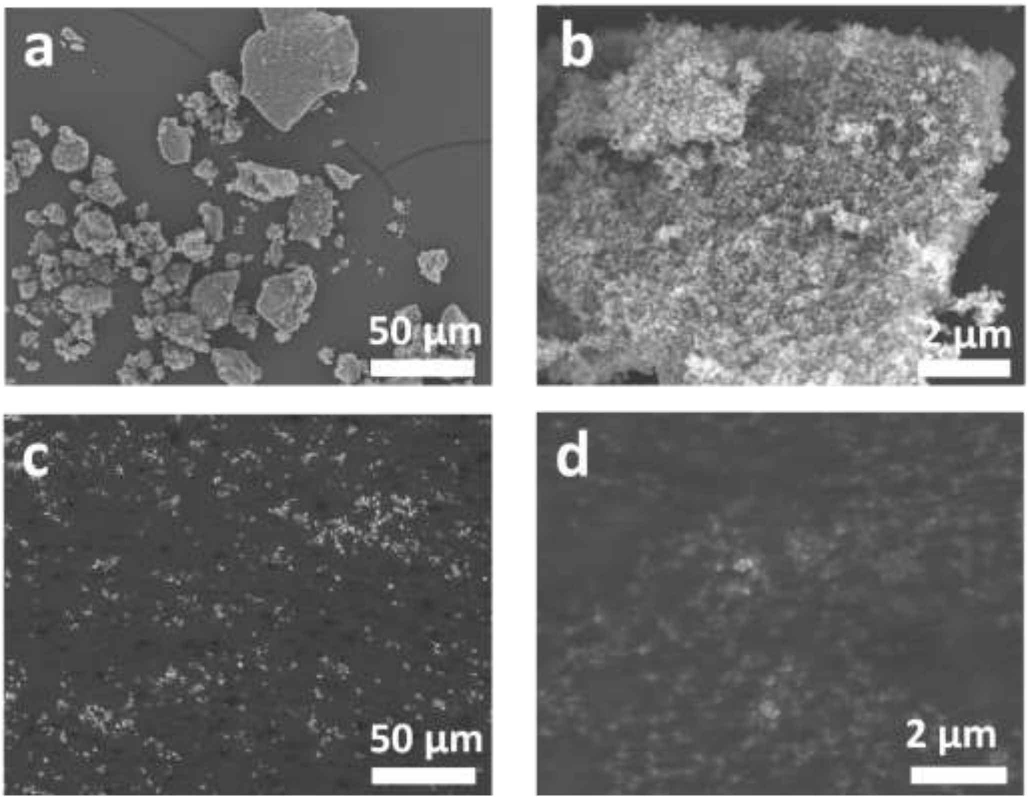

Figure S2. SEM images of (a) and (b), the pristine shell nanomaterial agglomerates, (c) and (d), the collected nano-vapor of shell nanomaterials by tape.

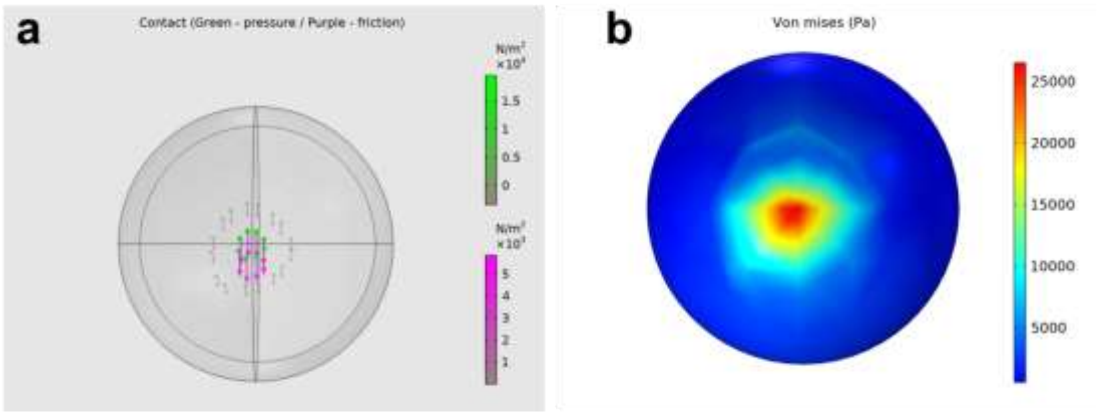

Figure S3. COMSOL simulation of the friction force (a), and stress distribution for S-rich particle surface during friction, (b). Note: during the wall friction, the contact stress keeps almost unchanged after reaching steady state. Specifically, the stress and friction are～2.5×10^{-4} Pa and ～5×10^{-3} Pa, respectively.

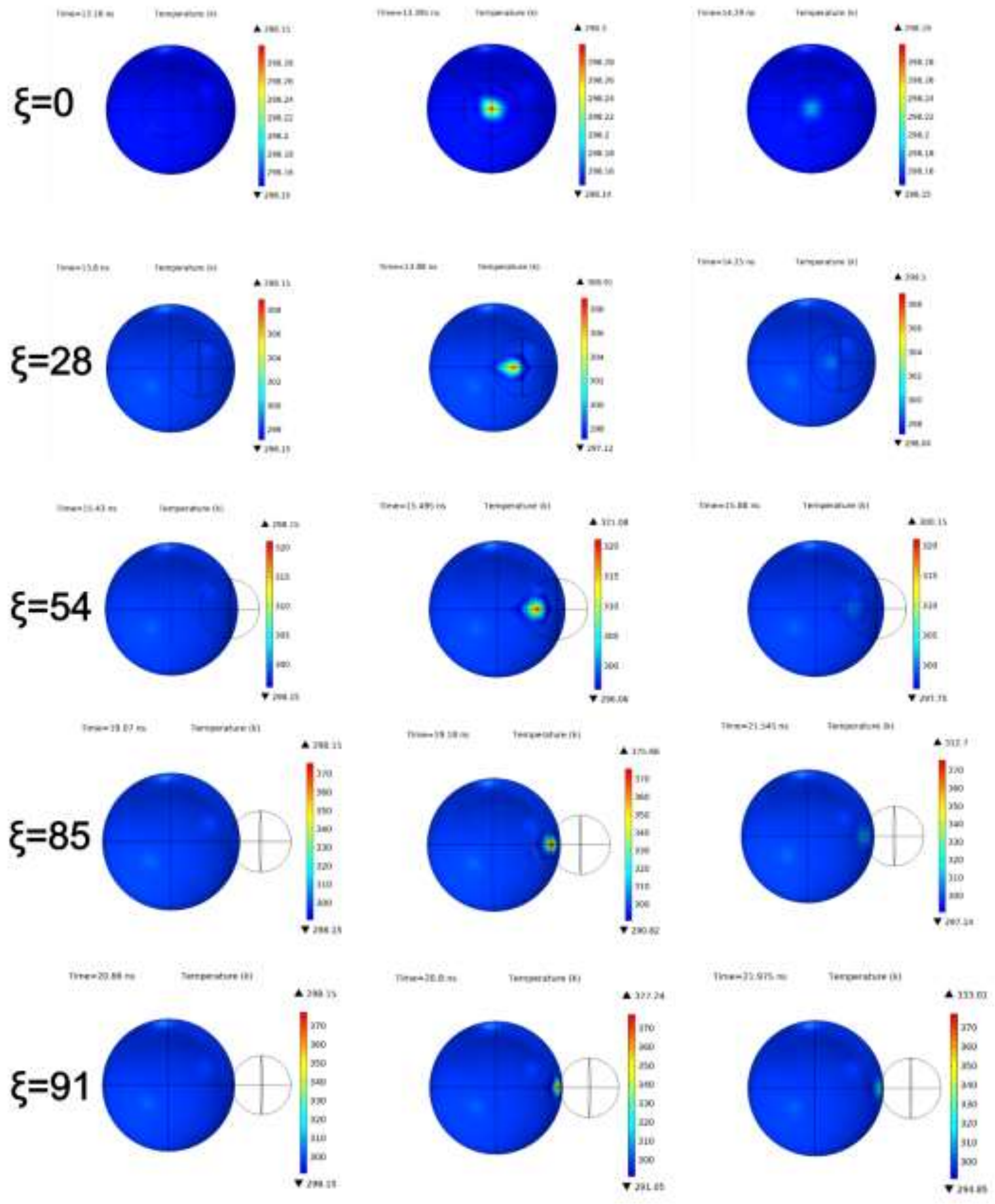

Figure S4. COMSOL simulation of the temperature rise at the collision point by friction between S/C particle and the shell nanomaterials under various eccentricities (ξ).

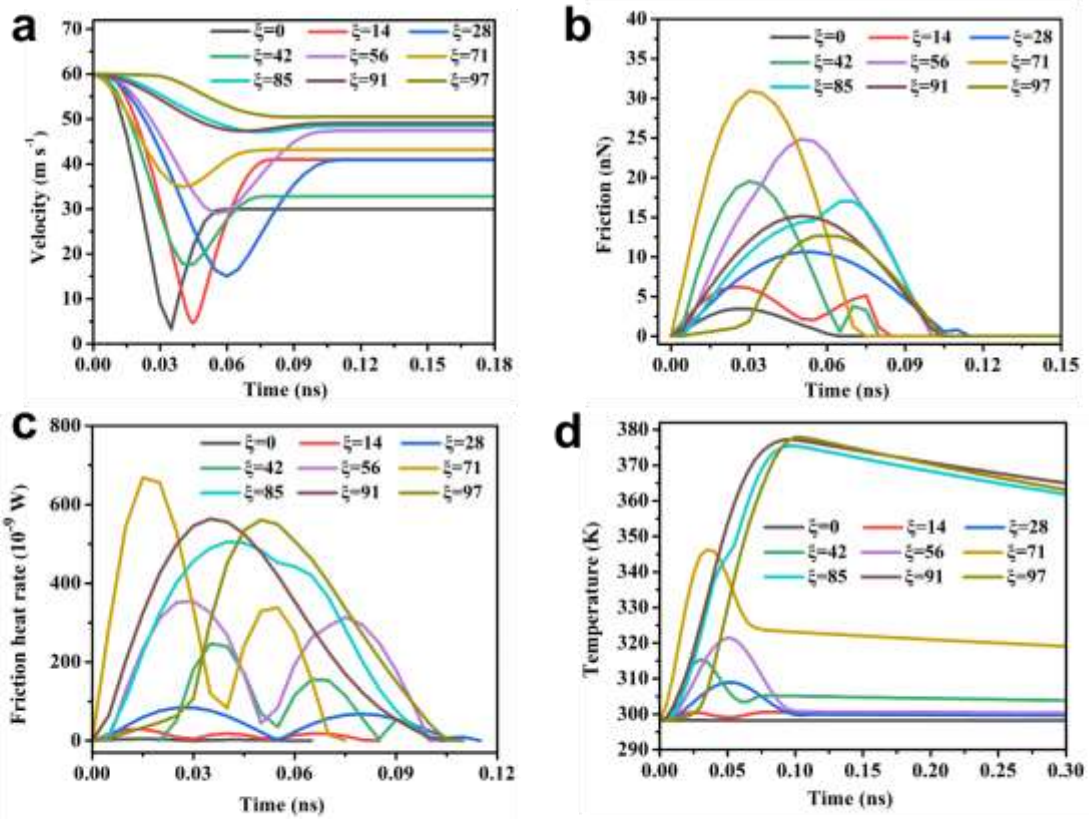

Figure S5. COMSOL simulation of the tendency of (a) carbon nanoparticle velocity and (b) friction under various eccentricities of collision friction. (c) The calculated friction heat rate for various eccentricities of collision friction from carbon nanoparticle velocity and friction. (d) Comparison of the temperature rising behavior for sulfur surface under various eccentricities of collision friction.

Note: the integral product of velocity and friction represents the friction heat rate in our work. Furthermore, the heat energy is calculated from the integral of friction heat rate. From the Figure S3c, bigger eccentricity (ξ), we have defined in Figure 2b, not only has bigger friction heat rate, but also possesses longer friction time, thus leading to a higher temperature rising (see Figure 2b and Figure S3d).

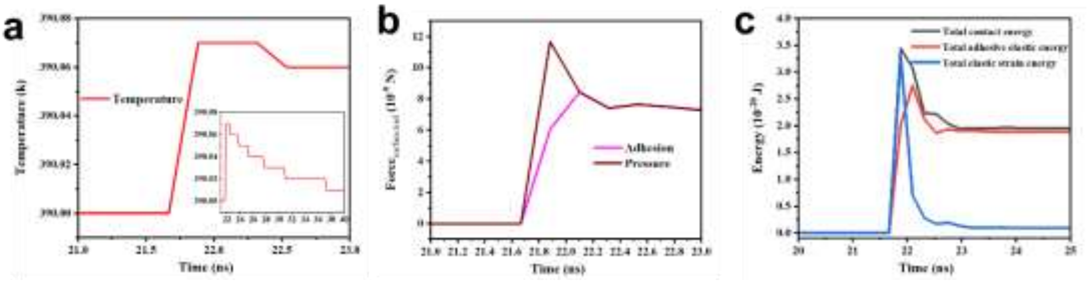

Figure S6. COMSOL simulation of (a) velocity change for carbon nanomaterial, (b) temperature change and (c) total adhesion force and pressure for sulfur surface during adhesive collision.

Note: during the adhesive collision, the velocity of carbon nanoparticle slowly decreases due to the inertial effect (see Figure 2d). Therefore, the temperature decrease shows a plateau, as shown in Figure S4a. Besides, Figure S4b shows that the value of adhesion and pressure converges within the process of collision. The possible explanation is that adhesion and pressure form counter force with the friction disappearing. For the adhesive collision, we think that $E_{\text{total contact energy}} = E_{\text{adhesive elastic energy}} + E_{\text{friction energy}} + E_{\text{elastic strain energy}}$. From the Figure S4c, we can observe that $\mathrm{E}_{\text{friction energy}}$ is negligible, and most of $E_{\text{total contact energy}}$ is dissipated by $E_{\text{adhesive elastic energy}}$.

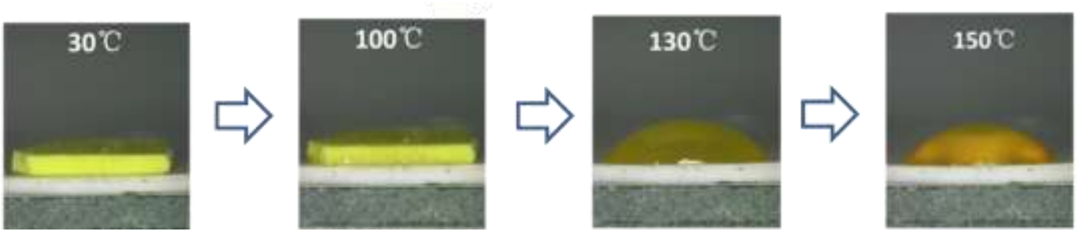

Figure S7. The melting of sulfur block when heated from 30 °C to 150 °C.

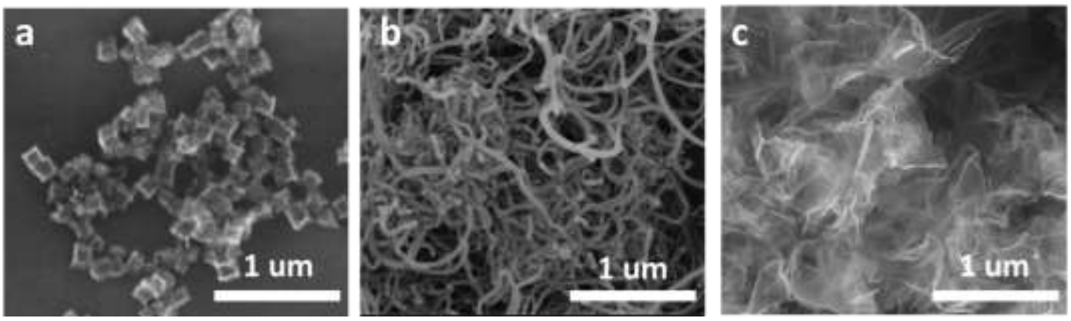

Figure S8. SEM images of shell nanomaterials (a) PNC, (b) CNT and (c) RGO.

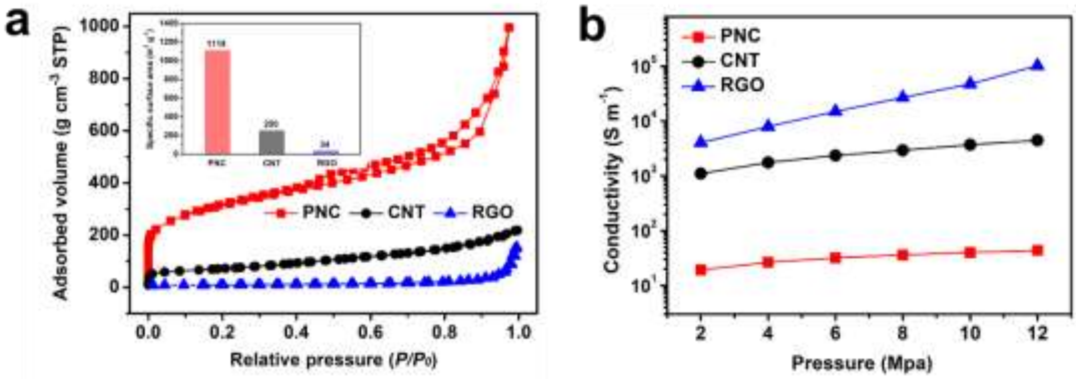

Figure S9. (a) N_2 adsorption–desorption isotherms of PNC, CNT and RGO shell nanomaterials, inset: specific surface areal comparison. (b) Comparison of the conductivities of PNC, CNT and RGO powders under varying pressure.

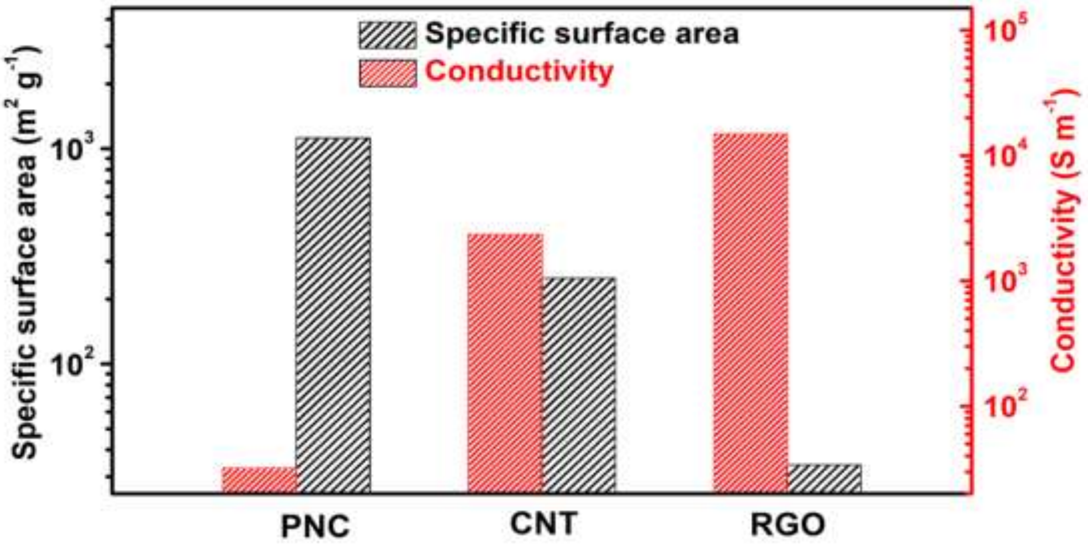

Figure S10. Comparison of the conductivity and specific surface area for PNC, CNT and RGO shell nanomaterials.

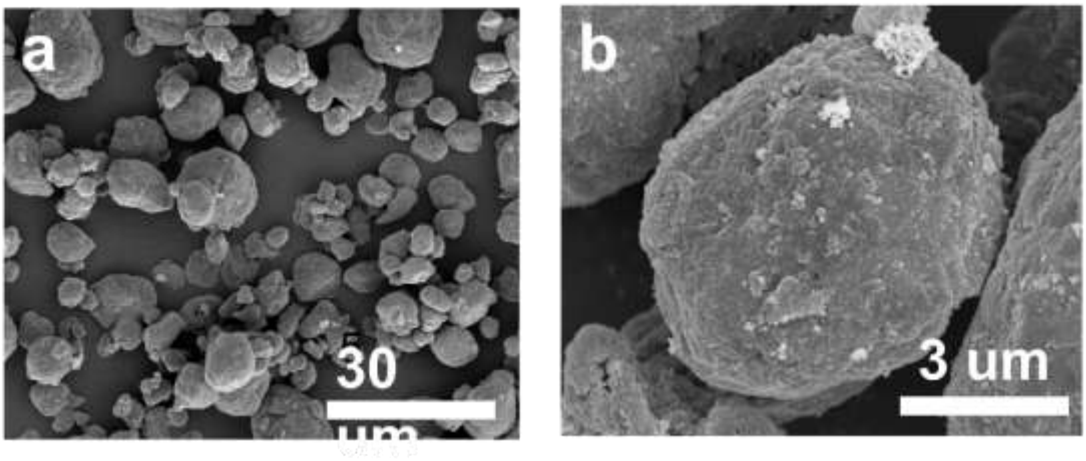

Figure S11. SEM images of pristine SC particles.

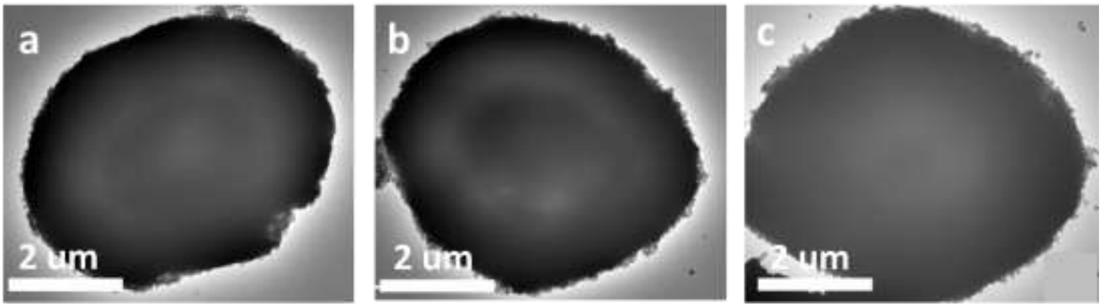

Figure S12. TEM images of the entire core-shell PNC@SC particles with different loading of PNC from (a) 1PNC@SC, (b) 3 wt% 3PNC@SC and (c) 5 wt% (5PNC@SC), respectively.

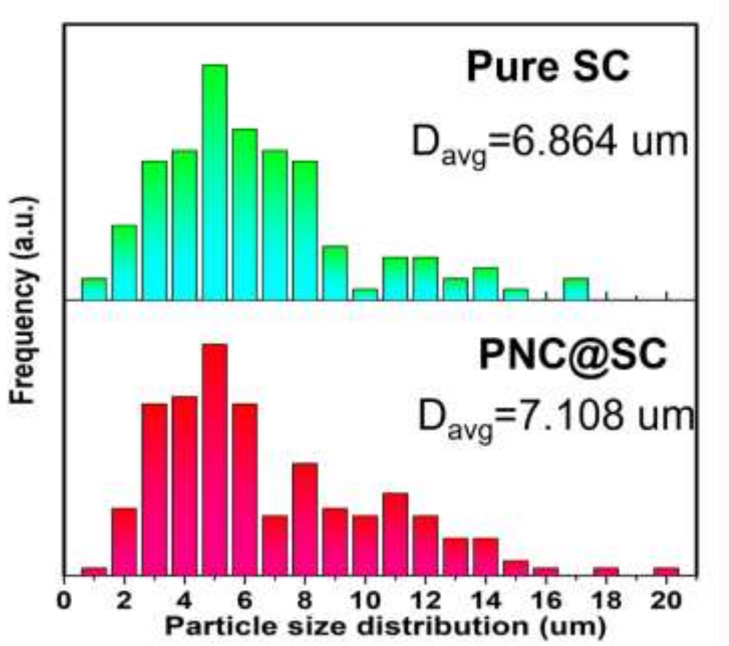

Figure S13. Size statistics comparison between the pure SC and 3PNC@SC particles.

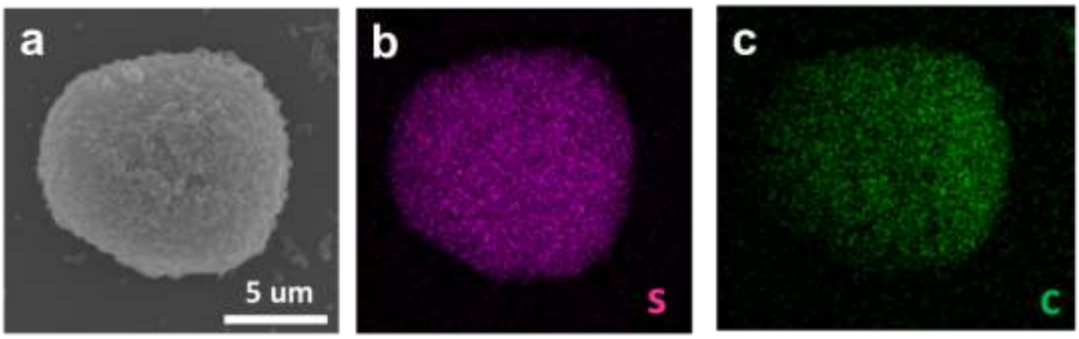

Figure S14. (a) EDS image of a single 3PNC@SC particle and its (b) S and (c) C element mapping.

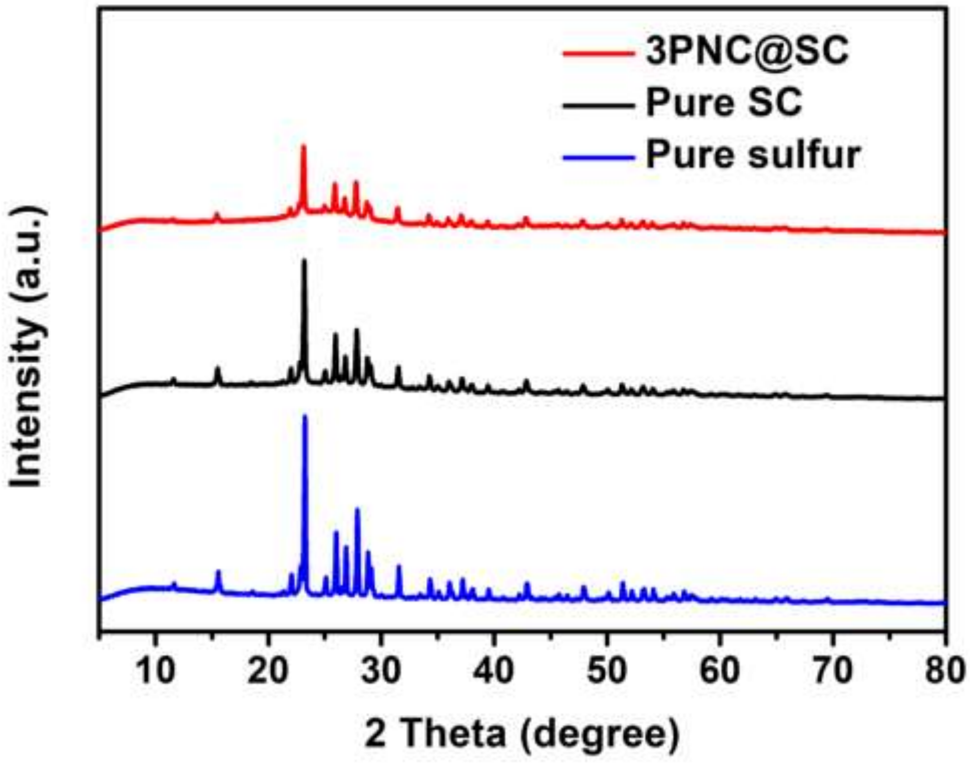

Figure S15. XRD patterns of pure sulfur, pure SC and 3PNC@SC.

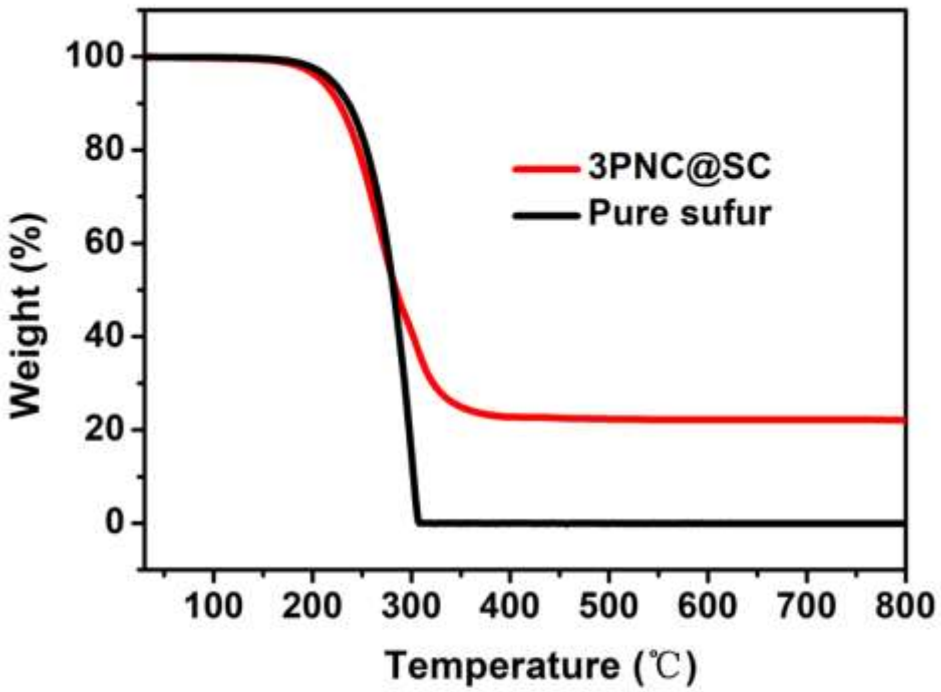

Figure S16. TGA curves of pure sulfur and the core-shell 3PNC@SC.

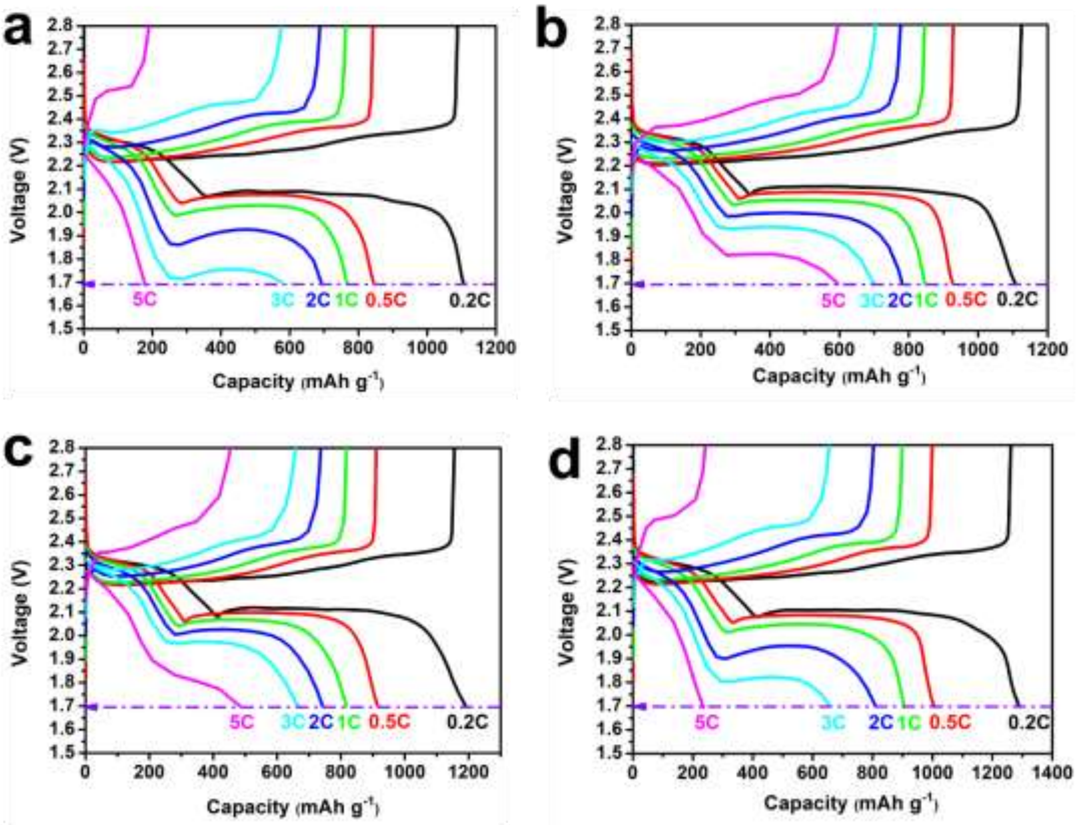

Figure S17. The galvanostatic discharge/charge profiles for (a) pure sulfur, (b) 1PNC@SC, (c) 3PNC@SC and (d) 5PNC@SC electrodes at various densities from 0.2C to 5C.

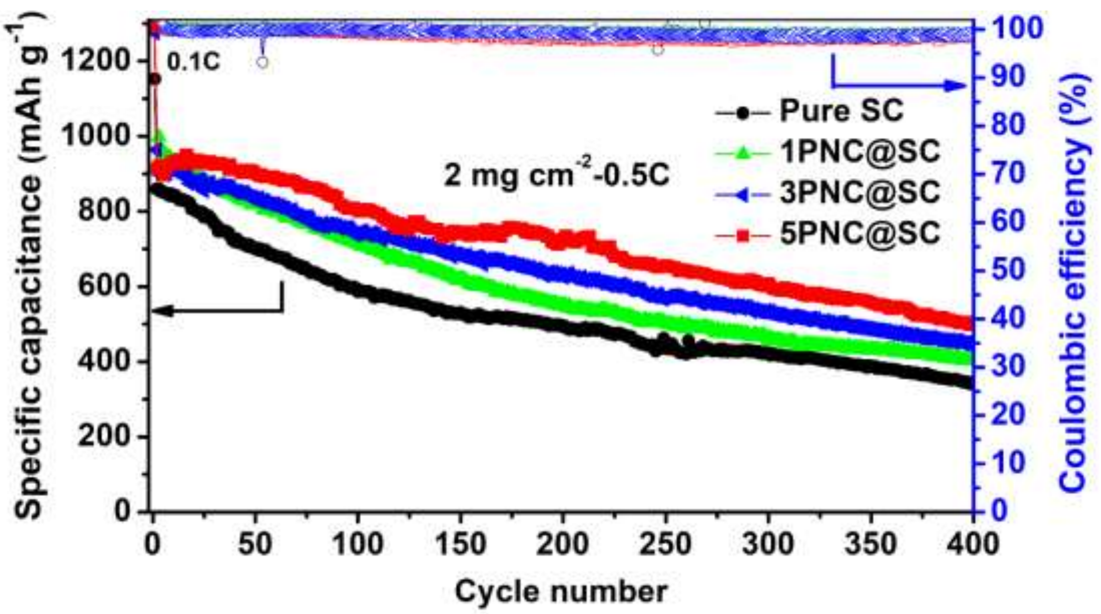

Figure S18. Comparison of cycle performance of pure SC, 1PNC@SC, 3PNC@SC, and 5PNC@SC electrodes at 0.5C.

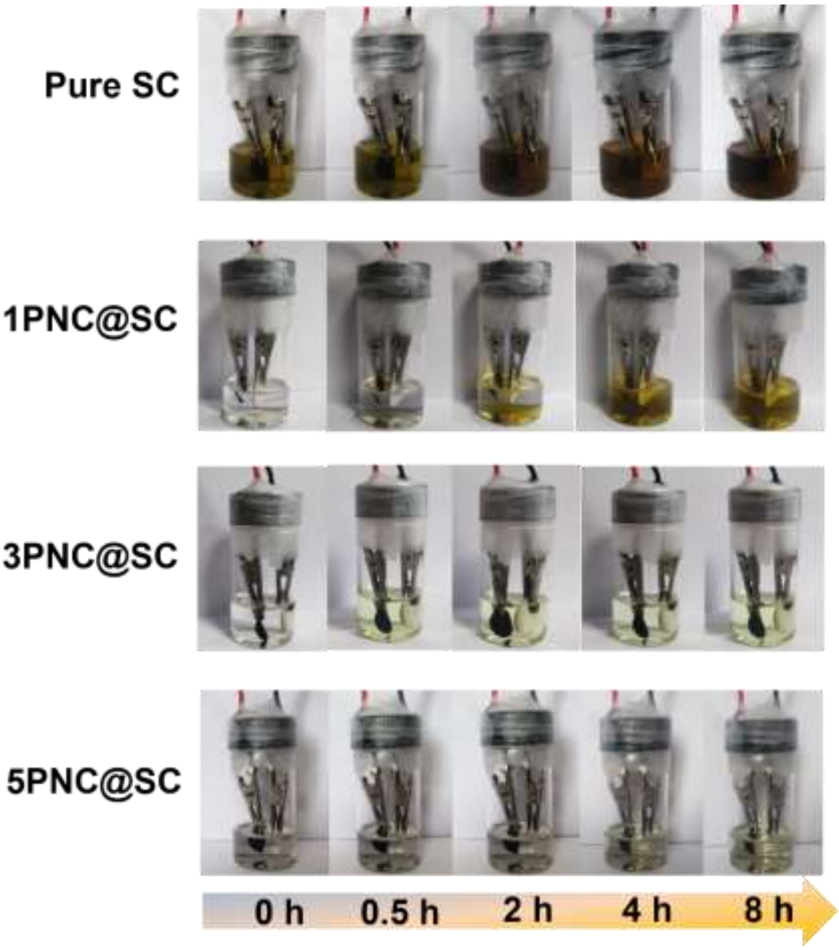

Figure S19. Polysulfide diffusion testing in open cells for the core-shell PNC@SC electrodes with various PNC shell thickness during discharging at 0.05 C.

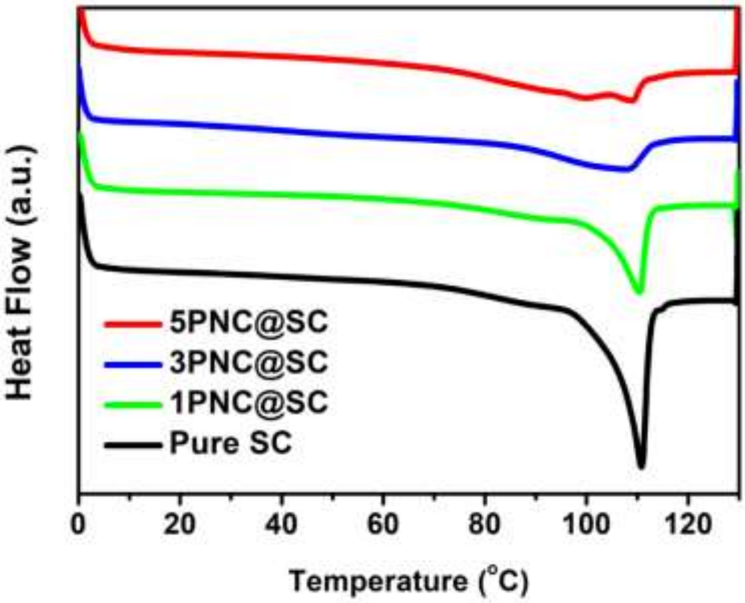

Figure S20. The melting behavior of the core-shell PNC@SC electrode particles

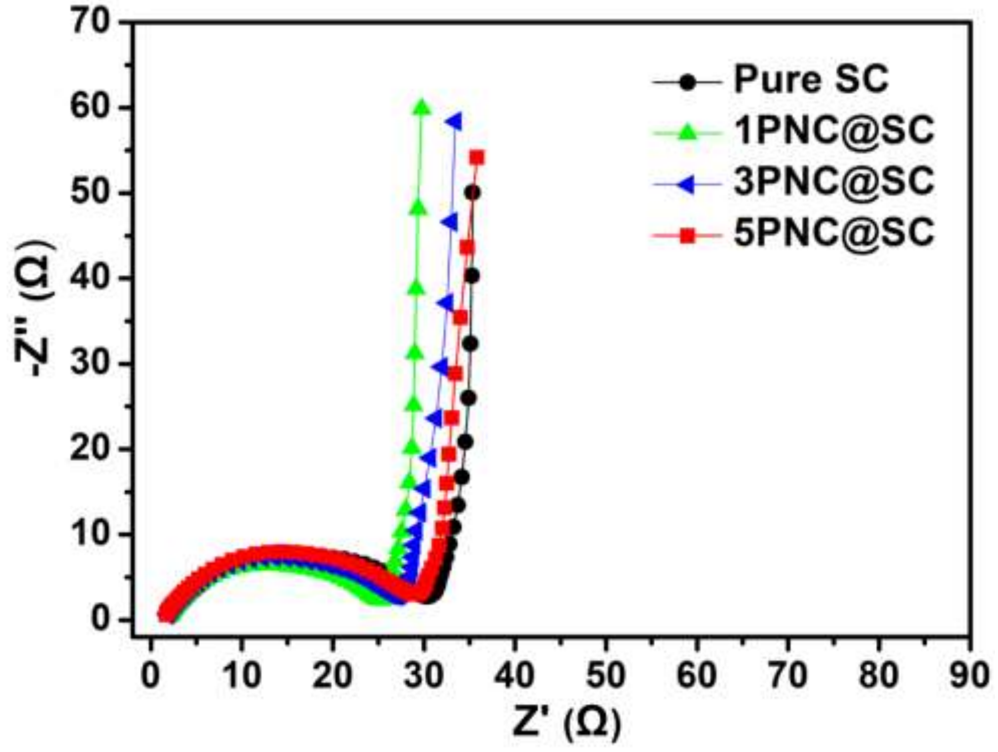

Figure S21. Nyquist plots of the electrodes based on the core-shell PNC@SC with different shell thickness.

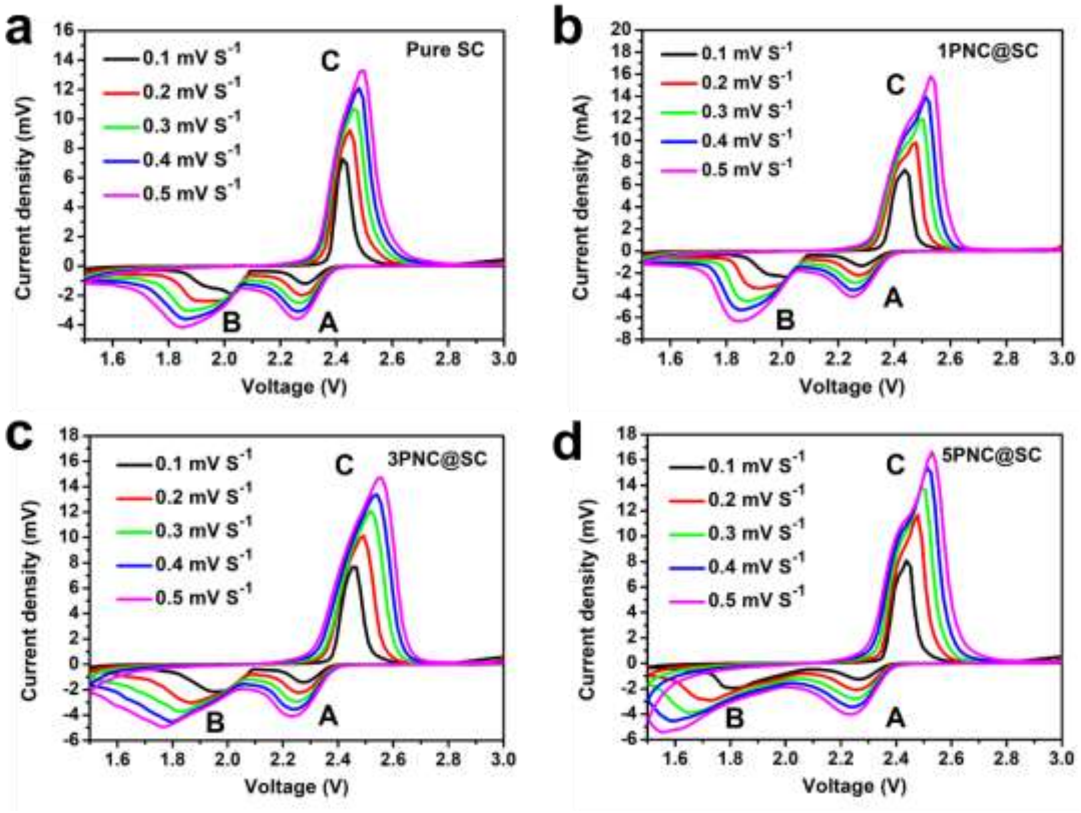

Figure S22. CV profiles of (a) pure sulfur, (b) 1PNC@SC, (c) 3PNC@SC and (d) 5PNC@SC electrodes at various scanning rates from 0.1 mV s^{-1} to 0.5 mV s^{-1}.

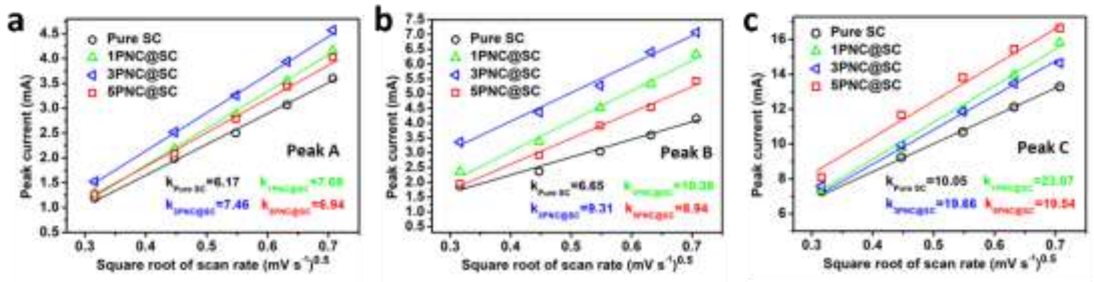

Figure S23. The linear fits of peak currents at (a) A, (b) B and (c) C peaks versus square root of scan rate from the CV scanning testing with scanning rates from 0.1 to 0.5 mV s^{-1}.

Table S1. Comparisons of the linear fitting values of Li^{+} diffusion coefficient for PNC@SC cathode with various thickness of PNC shell.

	D(α) (10^{-7} cm^2/s)	D(β) (10^{-7} cm^2/s)	D(γ) (10^{-7} cm^2/s)
Pure SC	0.54114	0.48838	4.09236
1PNC@SC	0.77924	1.47325	6.62895
3PNC@SC	0.77924	1.30009	5.03394
5PNC@SC	0.71566	1.13774	5.03394

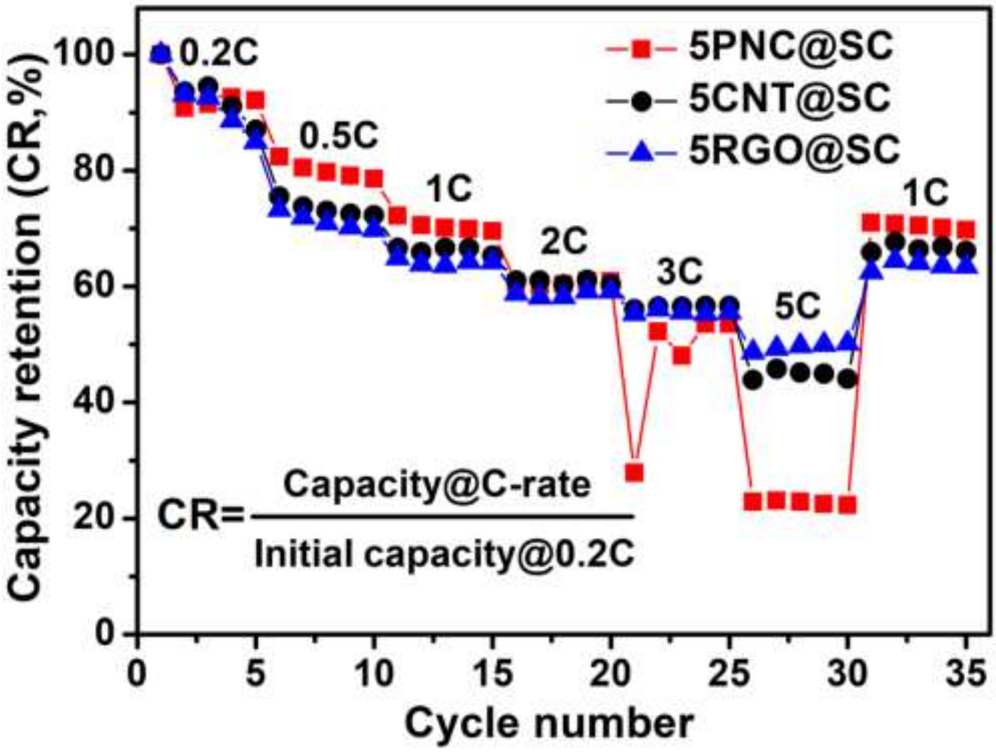

Figure S24. Capacity retention capability at different C-rates for 5PNC@SC, 5CNT@SC and 5RGO@SC electrodes.

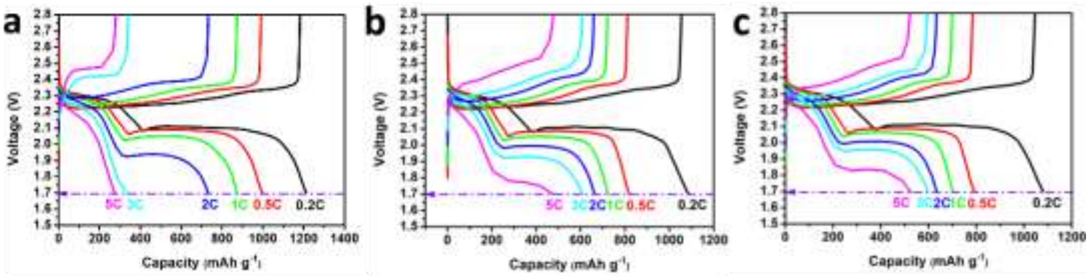

Figure S25. The galvanostatic discharge/charge profiles for (a) 5PNC@SC, (b) 5CNT@SC, (c) 5RGO@SC and electrodes at various densities from 0.2C to 5C.

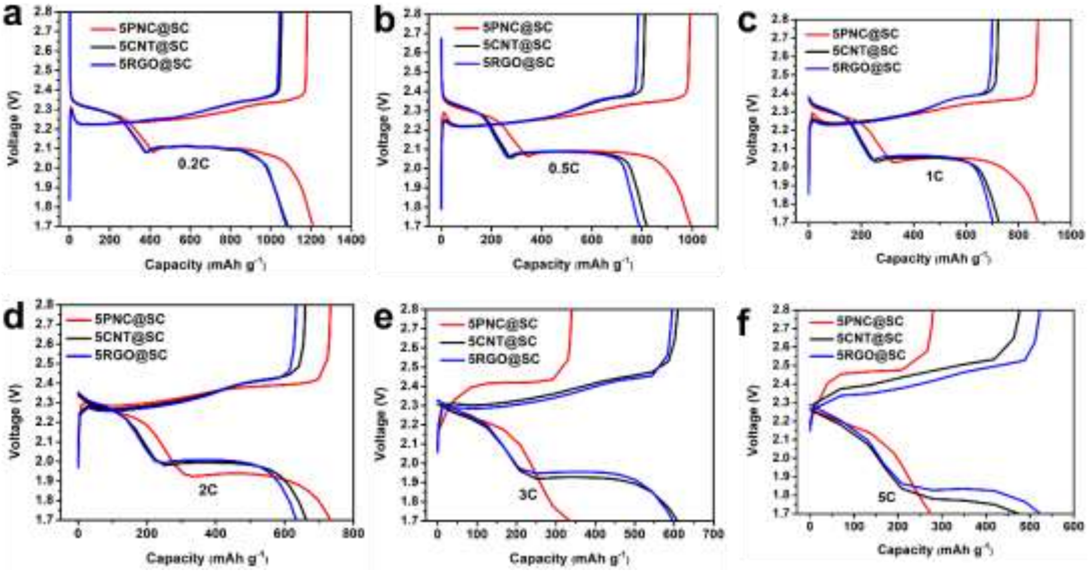

Figure S26. Comparison of galvanostatic discharge/charge profiles for 5PNC@SC, 5CNT@SC, and 5RGO@SC electrodes at various densities from 0.2C to 5C.

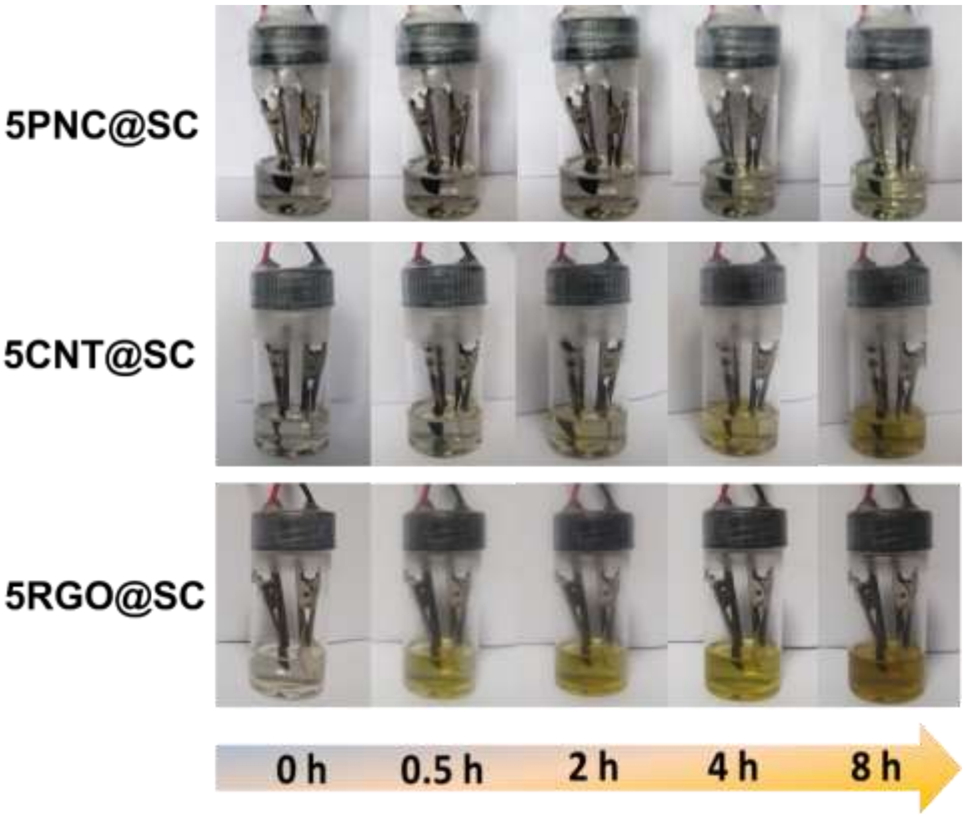

Figure S27. Polysulfide diffusion testing in open cells for the 5PNC@SC, 5CNT@SC and 5RGO@SC electrodes during discharging at 0.05 C.

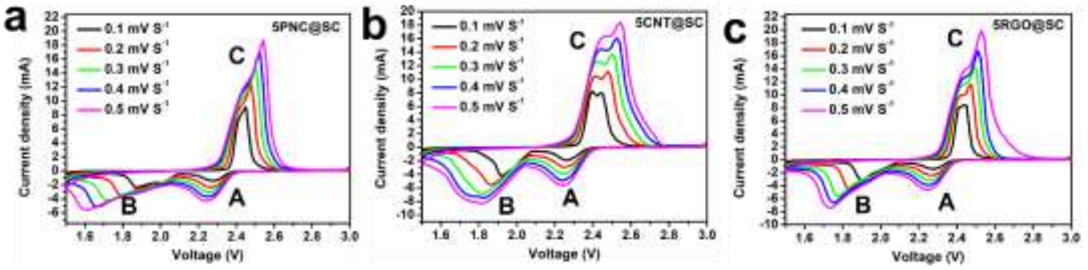

Figure S28. CV profiles of (a) 5PNC@SC, (b) 5CNT@SC and (c) 5RGO@SC electrodes at various scanning rates from 0.1 mV s^{-1} to 0.5 mV s^{-1}.

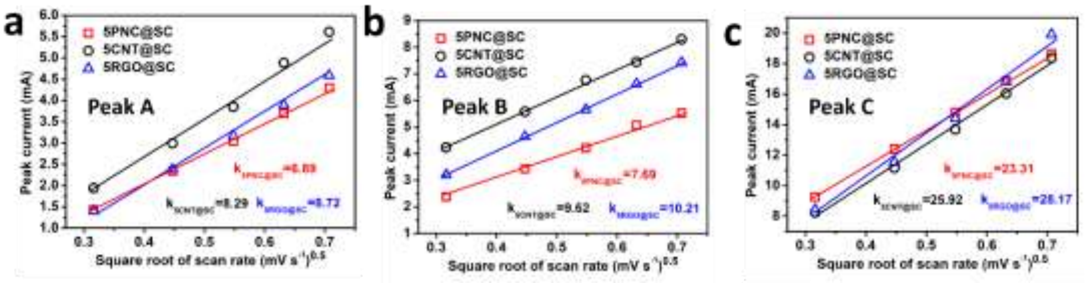

Figure S29. The linear fits of (a) peak A, (b) peak B and (c) peak C versus square root of scan rate from the CV testing.

Table S2. Comparisons of the linear fitting values of Li^+ diffusion coefficient for 5PNC@SC, 5CNT@SC and 5RGO@SC cathodes.

	D(α) (10^{-7} cm^2/s)	D(β) (10^{-7} cm^2/s)	D(γ) (10^{-7} cm^2/s)
5PNC@SC	0.71566	0.98623	6.25556
5CNT@SC	1.13774	1.56389	9.54569
5RGO@SC	1.13774	1.65724	10.47644

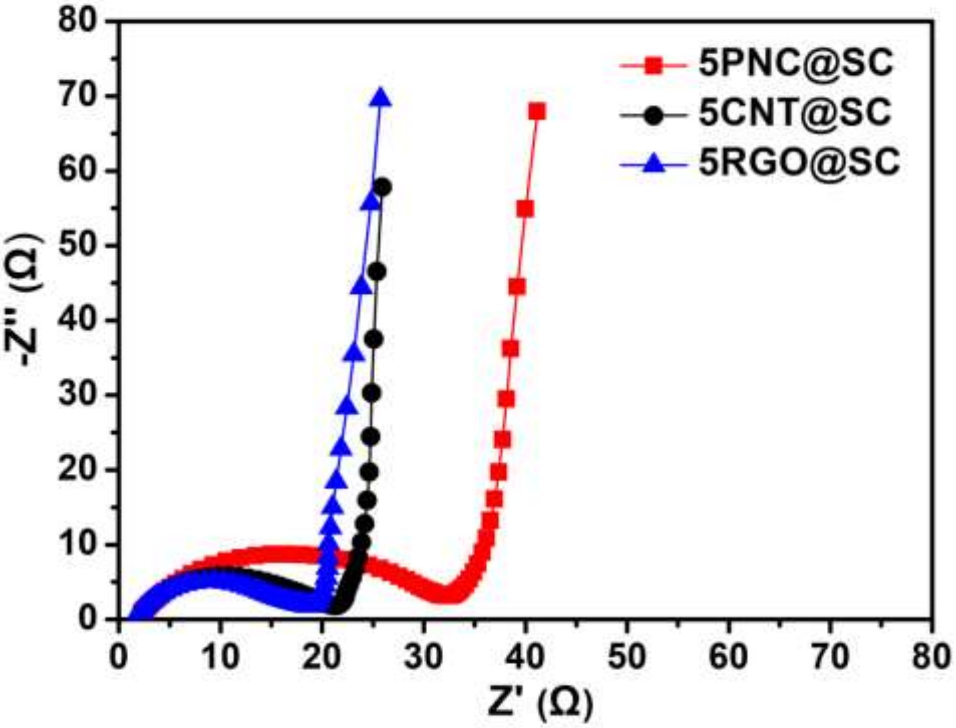

Figure S30. Nyquist plots of 5PNC@SC, 5CNT@SC and 5RGO@SC cathodes from electrochemical impedance spectroscopies.

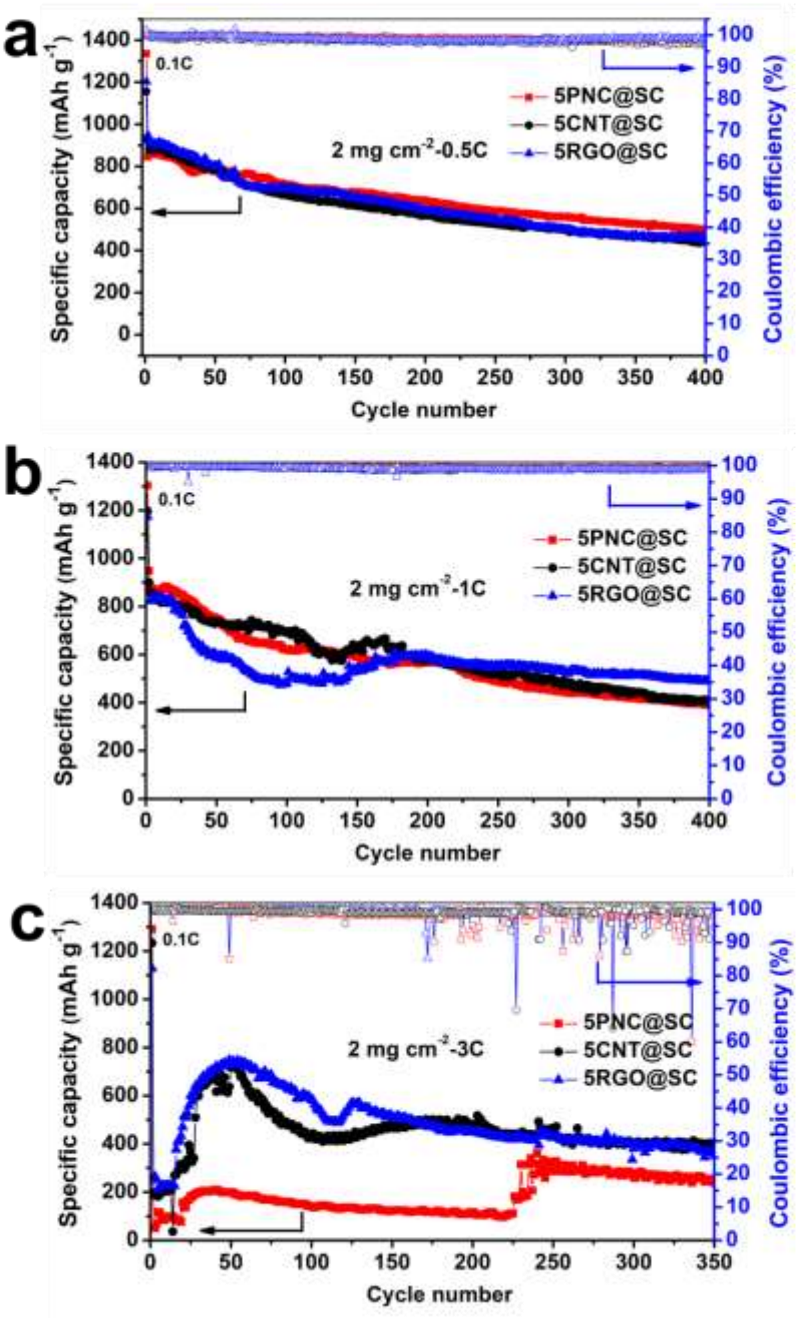

Figure S31. Comparison of the cycling performance for three types of core-shell sulfur based electrodes with a low sulfur loading of 2 mg cm^{-2} at (a) 0.5C, (b) 1C and (c) 3C, respectively.

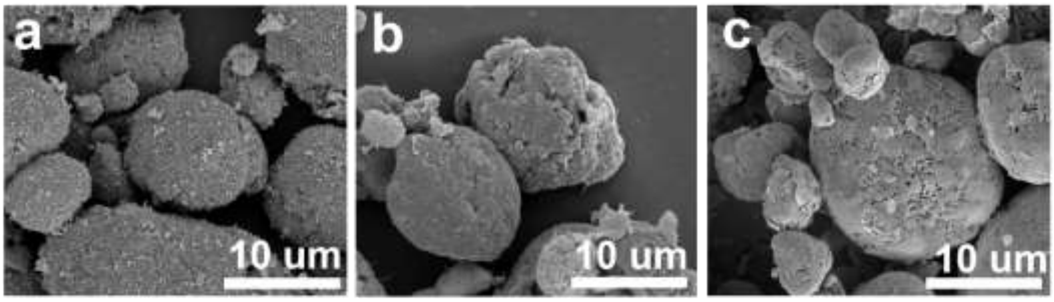

Figure S32. SEM images of the 5PNC@SC, (b) 5CNT@SC and (c) 5RGO@SC particles after vaccum treatment at 160 ℃ for 10 h.

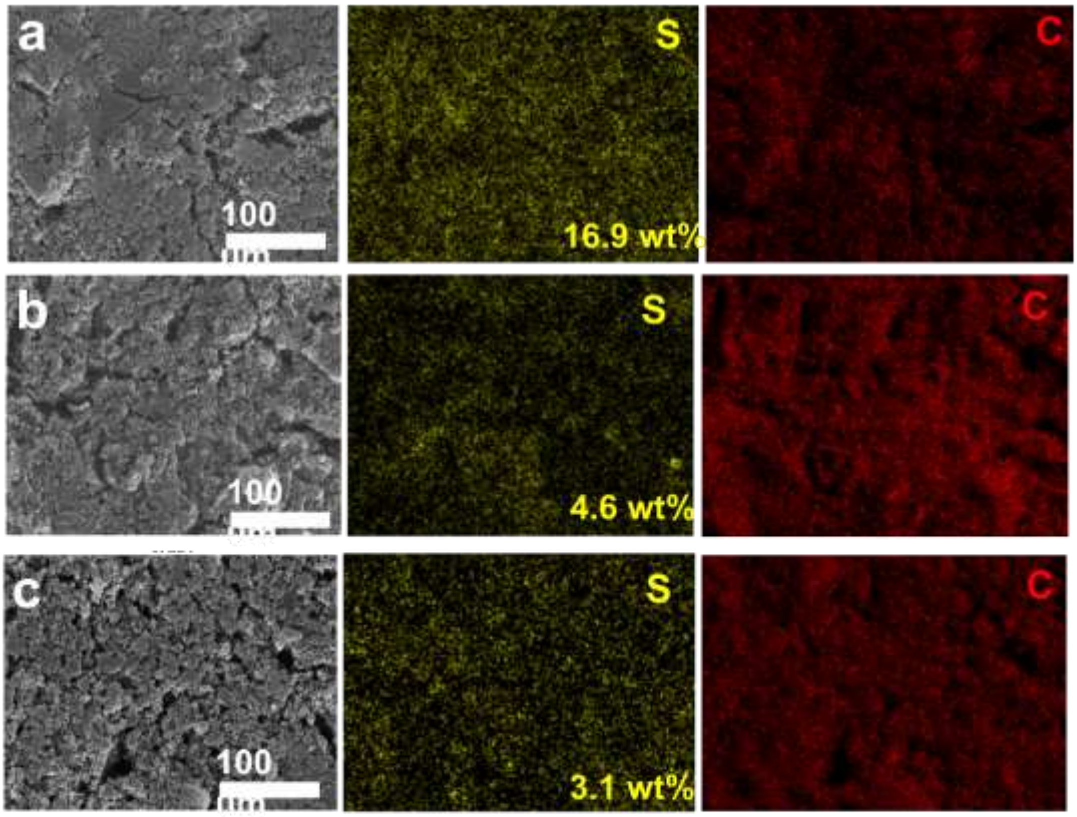

Figure S33. Figure S14. SEM and element mapping of cycled (a) 5PNC@SC, (b) 5CNT@SC and (c) 5RGO@SC cathodes.

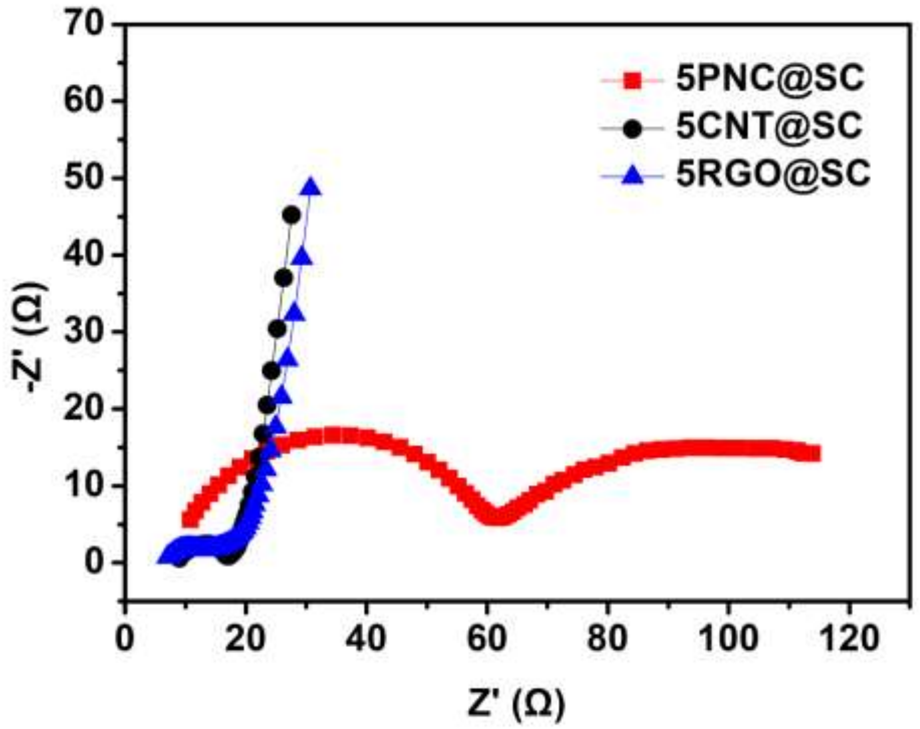

Figure S34. Nyquist plots of 5PNC@SC, 5CNT@SC and 5RGO@SC cathodes after 200 cycles from electrochemical impedance spectroscopies.

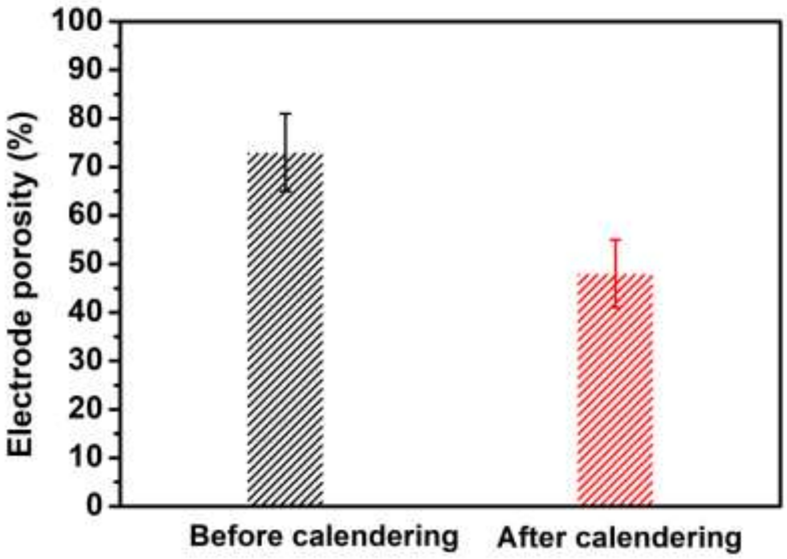

Figure S35. Comparison of the electrode porosity of 3PNC@SC cathode before and after calendering.

Reference

[1] M. Cui, B.-B. Xu, J. Lv, X.-W. Gao, Y. Zhang, *Int. J. Heat Mass Transfer* **2018**, 126, 1111.

[2] A. F. Bower, *Applied mechanics of solids*, CRC press, Boca Raton, FL, USA, **2009**.
